# Remote Control of Intestinal Stem Cell Activity by Haemocytes in *Drosophila*

**DOI:** 10.1371/journal.pgen.1006089

**Published:** 2016-05-27

**Authors:** Sveta Chakrabarti, Jan Paul Dudzic, Xiaoxue Li, Esther Jeanne Collas, Jean-Phillipe Boquete, Bruno Lemaitre

**Affiliations:** Global Health Institute, École Polytechnique Fédérale de Lausanne, Lausanne, Switzerland; University of California, Los Angeles, UNITED STATES

## Abstract

The JAK/STAT pathway is a key signaling pathway in the regulation of development and immunity in metazoans. In contrast to the multiple combinatorial JAK/STAT pathways in mammals, only one canonical JAK/STAT pathway exists in *Drosophila*. It is activated by three secreted proteins of the Unpaired family (Upd): Upd1, Upd2 and Upd3. Although many studies have established a link between JAK/STAT activation and tissue damage, the mode of activation and the precise function of this pathway in the *Drosophila* systemic immune response remain unclear. In this study, we used mutations in *upd2* and *upd3* to investigate the role of the JAK/STAT pathway in the systemic immune response. Our study shows that haemocytes express the three *upd* genes and that injury markedly induces the expression of *upd3* by the JNK pathway in haemocytes, which in turn activates the JAK/STAT pathway in the fat body and the gut. Surprisingly, release of Upd3 from haemocytes upon injury can remotely stimulate stem cell proliferation and the expression of *Drosomycin*-like genes in the intestine. Our results also suggest that a certain level of intestinal epithelium renewal is required for optimal survival to septic injury. While haemocyte-derived Upd promotes intestinal stem cell activation and survival upon septic injury, haemocytes are dispensable for epithelium renewal upon oral bacterial infection. Our study also indicates that intestinal epithelium renewal is sensitive to insults from both the lumen and the haemocoel. It also reveals that release of Upds by haemocytes coordinates the wound-healing program in multiple tissues, including the gut, an organ whose integrity is critical to fly survival.

## Author Summary

Innate immunity acts as the primary line of defense to overcome invading organisms. This response starts through sensing of microbe-specific molecules such as lipopolysaccharide and peptidoglycan by host receptors. Metazoans can also recognize signals that are associated with tissue damage and wounding, to then activate specific pathways involved in tissue repair or inflammation. Previous studies have suggested a link between JAK/STAT activation and tissue repair in *Drosophila*, but the mode of activation and the precise function of this pathway remain unclear. In this article, we analyze the role of the JAK/STAT pathway in the resistance to wounding in *Drosophila*. We show that this pathway contributes to fly resistance to wounding and bacterial infection. Mechanistically, injury induces the production of secreted molecules called Unpaireds by blood cells, which then activate the JAK/STAT pathway in the fat body (an organ equivalent to the vertebrate liver) and in the gut. One of the most surprising findings of our study is that Unpaireds released from blood cells can remotely stimulate intestinal stem cell proliferation. Thus, we uncover an unexpected interaction between circulating blood cells and intestinal stem cell proliferation that contributes to fly survival after injury.

## Introduction

Innate immunity provides the first line of defense against invading pathogens. This response is initiated by host pattern recognition receptors (PRRs), which sense specific and conserved motifs called microbe associated molecular patterns (MAMPs) found in microbes, but not in the host. MAMPs include lipopolysaccharide, peptidoglycan, lipoproteins, and CpG motifs of DNA [1]. Upon the recognition of MAMPs, PRRs activate signaling cascades that trigger the expression of immune effectors and regulators. Over the years, many PRRs, their ligands and the downstream signaling cascade components have been identified [2]. Metazoans have also acquired the capacity to recognize signals called damage-associated molecular patterns (DAMPs). DAMPs are associated with tissue damage and wounding, and activate specific pathways involved in tissue repair or inflammation [3]. As most infections are associated with tissue damage, signaling pathways induced by both DAMPs and MAMPs participate in a coordinated manner to mount an effective host defense response. Several DAMPs have been identified in mammals, including cellular components such as ATP, uric acid, nucleic acids and actin [4]. However, the pathways and effector mechanisms that are activated by DAMPs and how these pathways are integrated with other facets of the innate immune response are poorly understood.

The JAK/STAT signaling pathway plays a pivotal role during development and immunity in both mammals and insects [5,6]. It is activated upon binding of a secreted ligand to a receptor, leading to the recruitment of the Janus kinase (JAK), and the subsequent activation of the transcription factor STAT (Signal Transducer and Activator of Transcription). STAT, which is activated through phosphorylation, then translocates into the nucleus where it regulates the expression of target genes. While the genome of mammals encodes multiple combinatorial JAK/STAT pathways, only one canonical pathway is found in *Drosophila*. The *Drosophila* JAK/STAT pathway was originally identified through its role in embryonic segmentation [7]. It has three main cellular components: the receptor Domeless, the JAK Hopscotch, and the transcription factor STAT92E [8], and is activated by three secreted proteins of the Unpaired (Upd) family–Upd1, Upd2 and Upd3 [9]. Several studies have revealed a role of JAK/STAT in the insect systemic immune response. The first evidence for a role of the JAK/STAT pathway in insect immunity was the observation that bacterial infection triggers the phosphorylation and translocation of STAT92E in the fat body of the mosquito *Anopheles gambiae* [10]. Subsequent gene expression profiling studies identified a subset of *Drosophila* immune-responsive genes that are regulated by JAK/STAT upon injection of bacteria in the body cavity (referred to as a septic injury). This includes the genes encoding a complement-like protein *Tep2*, the cytokine *Diedel*, a JAK/STAT negative regulator *Socs36E*, and *Turandots* [11–16]. *Turandots* are *Drosophila*-specific genes of unknown function that are induced under various stress conditions, especially septic injury [17]. Studies done in larvae have shown that septic injury induces the release of the cytokine Upd3 from haemocytes, leading to the activation of the JAK/STAT pathway in the fat body [18].

In addition to its role in the humoral response, the JAK/STAT pathway also plays a pivotal role in *Drosophila* haematopoiesis. Constitutive activation of the pathway in haemocytes leads to a 5–20 fold increase in plasmatocyte numbers in larvae [19]. Many of the plasmatocytes differentiate into large flat cells called lamellocytes, which play a role in the encapsulation and destruction of aberrant self-tissue [20]. The JAK/STAT pathway also participates in the control of tumors or aberrant self in flies. The Upd3 ligand is expressed either at a wound site or by tissues with damaged basement membrane due to tumor outgrowth [20, 21]. Circulating Upd3 then triggers JAK/STAT activation in both haemocytes and fat body, resulting, among others, in an increased number of haemocytes, which contribute to wound healing or tumor reduction [20].

Although many studies have suggested a link between JAK/STAT activation and tissue repair, the mode of activation and the precise function of this pathway in *Drosophila* systemic immunity remain unclear. In this article, we analyze the role of the JAK/STAT pathway in the resistance to wounding and septic injury in *Drosophila*. By using previously generated *upd2* and *upd3* mutants [22], we show that this pathway contributes to fly resistance to wounding and bacterial infection. Our study reveals that haemocytes express the three Upds and that injury induces the production of Upd3, which then activates the JAK/STAT pathway in the fat body and in the gut. One of the most surprising findings of the study is that Upds released from haemocytes can remotely stimulate intestinal stem cell proliferation. Thus, we uncover an unexpected interaction between haemocytes and intestinal stem cell proliferation that contributes to fly survival after injury.

## Results

### Upd2 and Upd3 contribute to survival after septic injury

**Fig 1:**
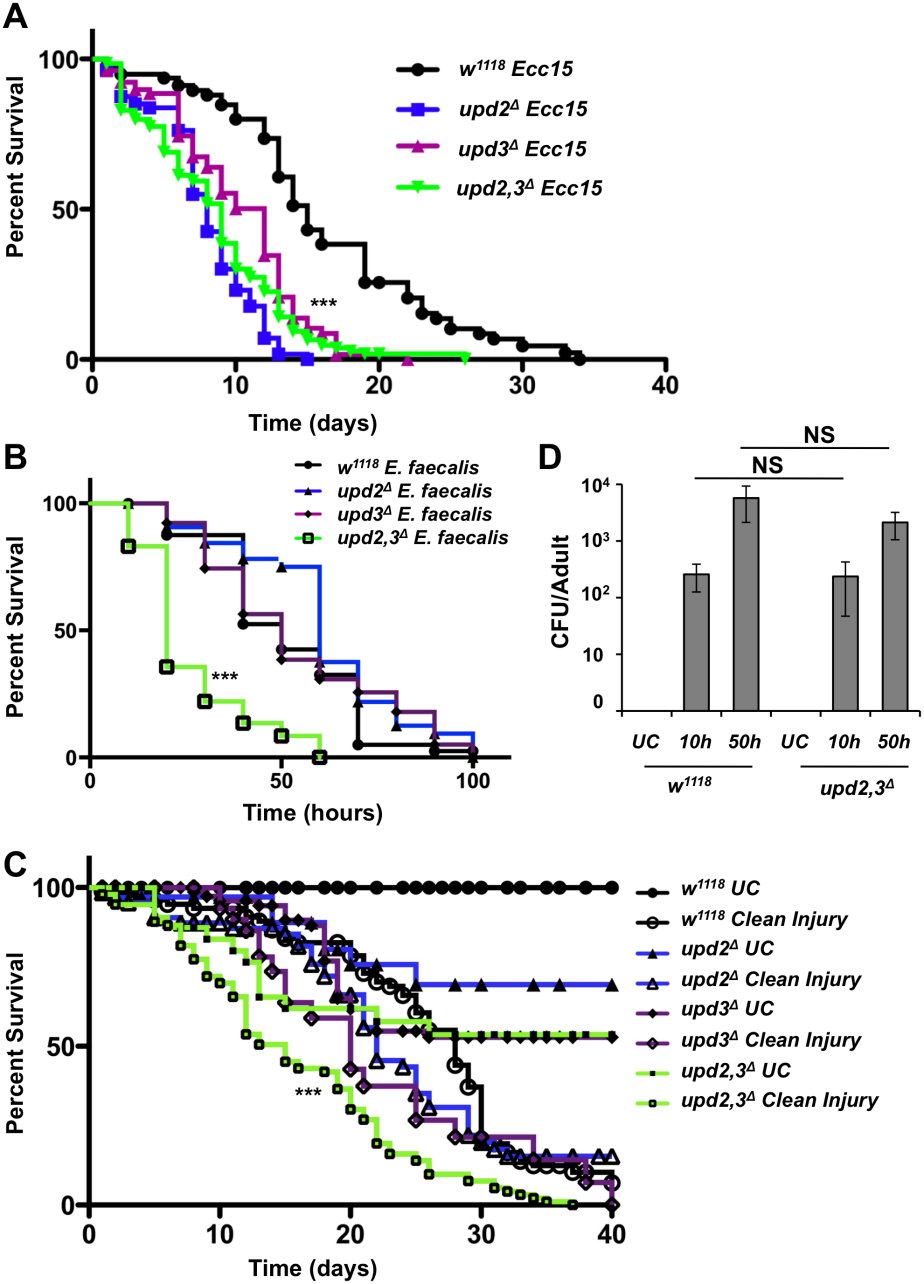
Upd ligands are required for the fitness of flies in response to septic injury with *Ecc15* and *E*. *faecalis*. (**A-B**) Survival analysis upon septic injury with *Ecc15* in (**A**) or *E*. *faecalis* in (**B**) of *upd2*^*Δ*^, *upd3*^*Δ*^ and *upd2*,*3*^*Δ*^ male flies as compared to wild-type (*w*^*1118*^). In (**A**) *upd2*^*Δ*^, *upd3*^*Δ*^ and *upd2*,*3*^*Δ*^ flies are more susceptible to infection with *Ecc15*. Flies were maintained at 29°C. Log-rank test comparing *upd2*^*Δ*^, *upd3*^*Δ*^ and *upd2*,*3*^*Δ*^ infected to *w*^*1118*^ infected: *** = p < 0.001. (**B**) *upd2*,*3*^*Δ*^ flies are more susceptible to infection with *E*. *faecalis*. Infections with *E*. *faecalis* were done at 25°C. Log-rank test comparing *upd2*,*3*^*Δ*^ infected to *w*^*1118*^ infected: *** = p, 0.0001. (**C**) Mortality of male flies injured with a clean needle in the thorax indicates that *upd2*,*3*^*Δ*^ flies are sensitive to wounding. Results in (**A-C**) are pooled data from at least four independent experiments, where 20 flies were used per genotype. Log-rank test comparing *upd2*,*3*^*Δ*^ injured to *w*^*1118*^ injured: *** = p < 0.001 (**D**) Bacterial persistence in wild-type and *upd2*,*3*^*Δ*^ male flies at 10 h and 50 h post septic injury with *E*. *faecalis* (OD_600_ = 10), expressed as the number of CFUs per fly. No difference was observed between *upd2*,*3*^*Δ*^ and *w*^*1118*^. Each histogram corresponds to the average of three independent experiments. Student’s *t* test shows P value = NS (0.93) for *upd2*,*3*^*Δ*^ vs *wt* flies at 10 h and P value = NS (0.31) for *upd2*,*3*^*Δ*^ vs *wt* flies at 50 h. NS: Non-Significant.

The *Drosophila* genome contains three *unpaired* genes that encode ligands capable of activating the JAK/STAT pathway receptor Domeless. Since *upd1* null mutants are embryonic lethal [8], we focused our attention on the role of *upd2* and *upd3* in the systemic immune response [22]. To assess their role in the antimicrobial response, we monitored the survival rate of flies lacking *upd2* and/or *upd3* after systemic infection with either the Gram-negative bacterium *Erwinia carotovora carotovora 15* (*Ecc15*) or the Gram-positive bacterium *Enterococcus faecalis*. Fig 1A shows that *upd2*^*Δ*^, *upd3*^*Δ*^ and *upd2*,*3*^*Δ*^ flies were significantly more susceptible than wild-type flies to septic injury with *Ecc15*. Compared to *Imd* deficient mutants, which usually die within 48h from the same challenge, the susceptibility of the *upd* mutants was mild with about 50% mortality after 10 days. Fig 1B shows that only *upd2*,*3*^*Δ*^ flies displayed a higher susceptibility to septic injury with *E*. *faecalis*. The absence of a differentiated survival phenotype for the *upd2*^*Δ*^ or *upd3*^*Δ*^ single mutants could be a result of the faster killing rate induced by this bacterium (Fig 1B).

The Toll and Imd pathways are two NF-kB signaling pathways that contribute to resistance to septic injury by regulating many immune genes, notably those encoding antimicrobial peptides [23]. One possibility is that loss of *upd2* and *upd3* disrupts one or both of these NF-kB pathways, resulting in lower survival to septic injury. To test this hypothesis, we monitored the expression of the antimicrobial peptide genes *Diptericin* (*Dpt*) and *Drosomycin* (*Drs*) as readouts of the Imd and Toll pathways, respectively, in *upd* mutants. Both antimicrobial peptide genes remain fully inducible in *upd2*^*Δ*^, *upd3*^*Δ*^ and *upd2*,*3*^*Δ*^ mutants (S1A and S1B Fig). This indicates that Upd ligands contribute to host survival to wounding and septic injury in a Toll and Imd independent manner.

At this stage, it was unclear whether the higher susceptibility of *upd* mutants to septic injury was caused by the presence of the bacterium itself or by the wounding associated with this mode of infection. To distinguish between the two, we monitored the survival rates of *upd* deficient flies to sterile wounding and indeed, *upd2*,*3*^*Δ*^ and to a lesser extent the single mutant flies showed an enhanced susceptibility to a clean wound (Fig 1C). We conclude that *upd2*,*3*^*Δ*^ flies are more susceptible to injury and that the presence of bacteria in the wound simply accelerates the mortality rate in both wild-type and *upd2*,*3*^*Δ*^ flies. Resistance and resilience mechanisms both contribute to the survival to bacterial infection, where resistance involves the activation of the immune responses to eliminate pathogens, whereas resilience encompasses the capacity to survive infection without eliminating the pathogen [24]. We observed that the number of *E*. *faecalis* in flies both 10 h and 50 h after infection (when flies start to die) was roughly similar in the *upd2*,*3*^*Δ*^ flies and in wild-type (Fig 1D). Hence, resistance mechanisms were unaffected in the *upd2*,*3*^*Δ*^ flies in response to bacterial infection. In the following step, we investigated how Upd ligands contribute to survival by restricting our analysis to two wounding protocols, clean or septic injury with *Ecc15* (referred to below as septic injury).

### Survival to wounding requires the production of Upd2 and Upd3 by haemocytes

**Fig 2:**
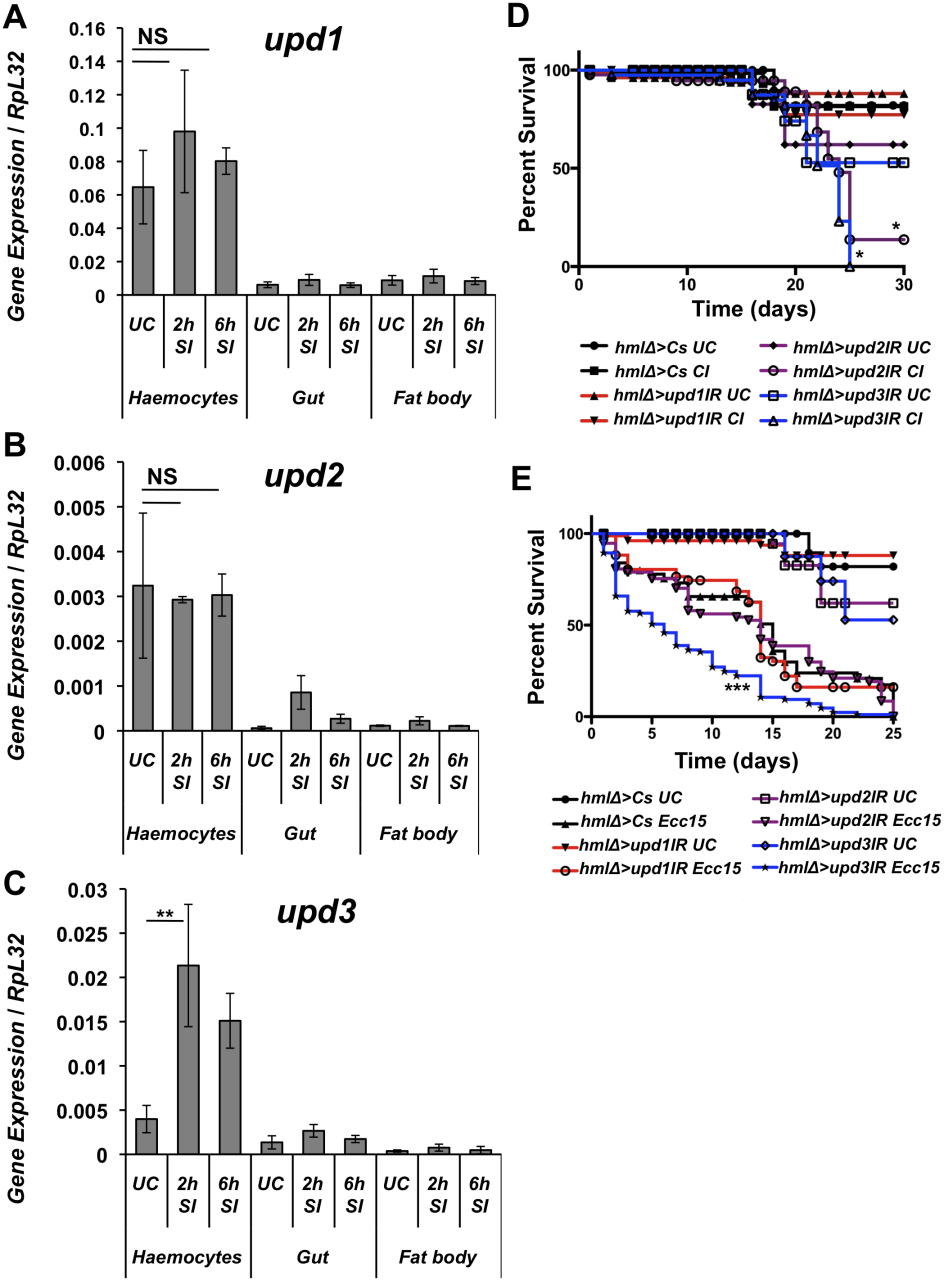
*Upd* genes are differentially expressed in the adult *Drosophila* haemocytes and midgut in response to septic injury. (**A**) RT-qPCR experiments show that *upd1* is expressed in haemocytes but its expression remains unchanged upon septic injury (SI). NS: P value = 0.479 between UC and 2 h SI for haemocytes. (**B**) *Upd2* is expressed in haemocytes in unchallenged flies (UC) but its expression remains unchanged upon septic injury (UC). NS: P value = 0.856 (**C**) *Upd3* is also expressed in haemocytes and its expression is increased upon septic injury. Mean values of at least three experiments ± SE are shown and data was analyzed by Student’s *t* test. (**D-E**) Survival analysis shows that flies with reduced *upd3* expression in haemocytes display an increased susceptibility to clean injury (CI) and septic injury. *RNAi* flies with reduced expression of *upd2* or *upd3* show higher mortality to clean injury **(D**) and only *upd3 RNAi* flies show a greater susceptibility to *Ecc15* septic injury (**E**). Log-rank test was used to determine statistical significance. Male flies were used for experiments.

Previous studies have shown that *upd1*, *upd2* and *upd3* are induced in various intestinal cell types upon oral infection to stimulate stem cell proliferation and differentiation. *Upd3* has also been shown to be induced in haemocytes of third instar larvae upon septic injury [15, 22, 25]. To investigate the spatial expression pattern of *upds* in adults, we monitored their expression in the three major immuno-responsive tissues, fat body, gut and haemocytes. In the haemocytes, *upd1* and *upd2* are expressed, but their expression is not consistently altered upon septic injury (Fig 2A and 2B). In contrast, the transcription of *upd3* is significantly up-regulated in haemocytes 2h post-infection (Fig 2C). In the gut, all three *upds* are expressed at basal levels. However, the expression of *upd2* was up-regulated at 2h following septic injury (Fig 2B). In contrast, the fat body did not significantly up-regulate any of these three genes (Fig 2A–2C). We next investigated whether Upds are required in adult haemocytes for optimal survival to wounding. For this, we knocked down individually each *upd* gene in haemocytes using an *in vivo RNAi* approach with the haemocyte driver *hmlΔGAL4* [26, 27] and monitored the survival of flies to wounding as well as to septic injury. We discovered that knockdown of either *upd2* or *upd3* in haemocytes increased the susceptibility of flies to wounding, while only the knockdown of *upd3* resulted in higher susceptibility to septic injury with *Ecc15* (Fig 2D and 2E). To reinforce our conclusion, we performed survival to clean and septic injury in flies that express both the *upd2* and *upd3 RNAi* using another GAL4 driver, *peroxidasin (pxn)GAL4* (S1C Fig). While *hmlΔGAL4* is a haemocyte specific driver [26, 27], *pxnGAL4* is expressed in haemocytes and weakly expressed in the fat body [28]. Simultaneous silencing of *upd2* and *upd3* with *pxnGAL4* markedly increased susceptibility to both clean and septic injury (S1C Fig). These experiments support the notion that the release of Upds by haemocytes promotes host survival to clean and septic injury. In agreement with this, flies lacking most of their haemocytes due to the expression of the pro-apoptotic factor Bax in the plasmatocyte lineage (genotype: *hmlΔGAL4 > UAS-Bax)* were susceptible to both wounding and septic injury showing similar survival kinetics (S1D Fig). Collectively, these results provide strong evidence that the production of Upd2 and Upd3 ligands by haemocytes is crucial for fly survival to wounding and septic infection.

### Septic injury activates JAK/STAT transcriptional activity in the gut

**Fig 3:**
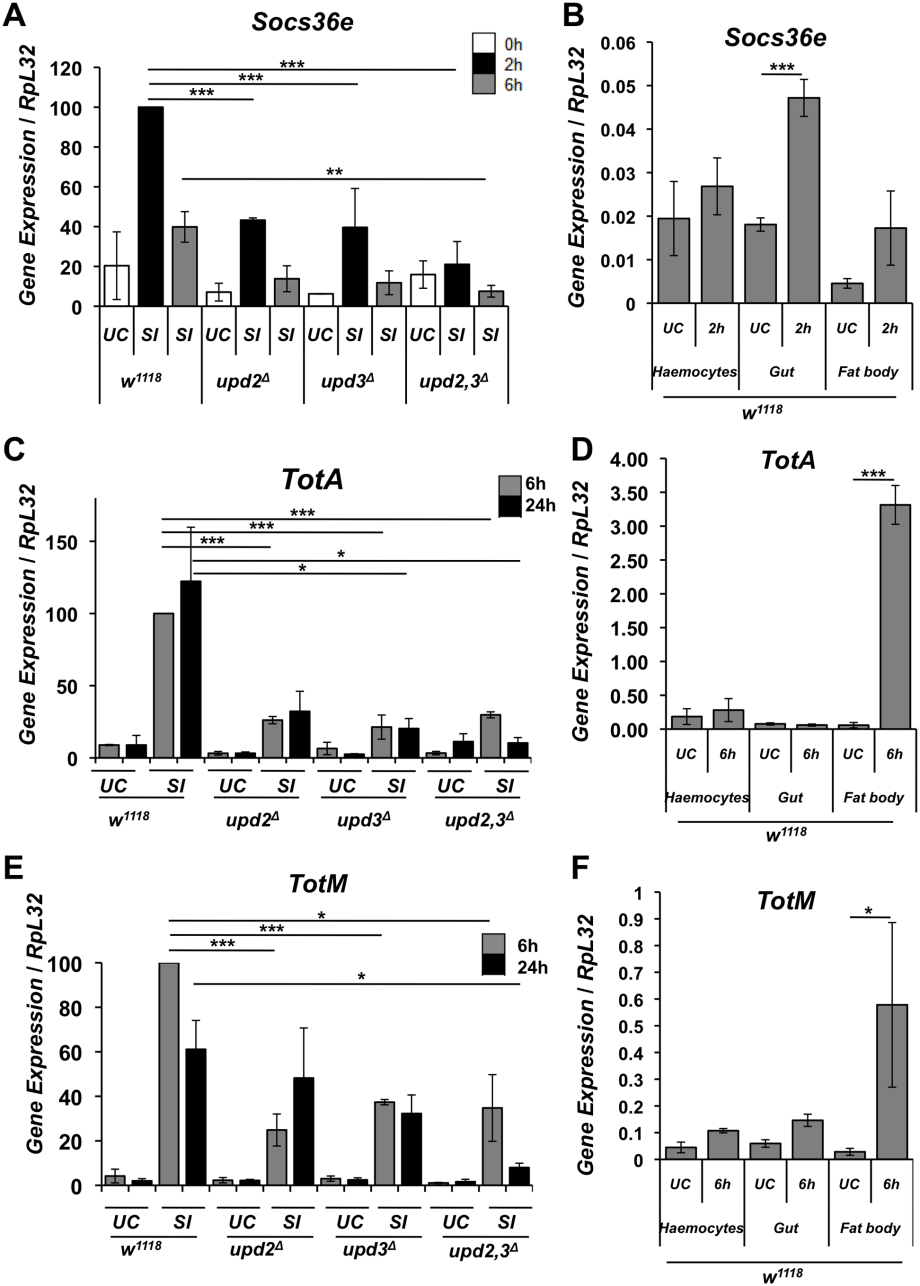
Upd ligands regulate JAK/STAT transcriptional activity in various tissues upon septic injury. (**A**) RT-qPCR experiments reveal that *upd2* and *upd3* regulate the expression of the JAK/STAT target gene *Socs36E* in adult flies in response to septic injury (SI) with *Ecc15*. (**B**) Tissue specific expression in *w*^*1118*^ flies by RT-qPCR experiments shows that *Socs36E* is up-regulated in the gut 2h after septic injury with *Ecc15*. (**C and E**) Expression of *TotA* and *TotM* genes in *upd2*^*Δ*^, *upd3*^*Δ*^ and *upd2*,*3*^*Δ*^ adult flies as compared to wild-type (*w*^*1118*^) after SI with *Ecc15*. A time course of *TotA* and *TotM* expression 6 h and 24 h after infection shows that *upd2* and *upd3* control the expression of *TotA* and *TotM*. (**D and F**) RT-qPCR in wild-type flies show that *TotA* and *TotM* are mostly induced in the fat body following septic infection. All RT qPCR analysis was done using male adult flies. Mean values of at least three experiments (with 30 to 40 flies each) ± SE are shown. ***: p< 0.001, **: p< 0.01, *: p< 0.05 as determined by Student’s t-test.

**Fig 4:**
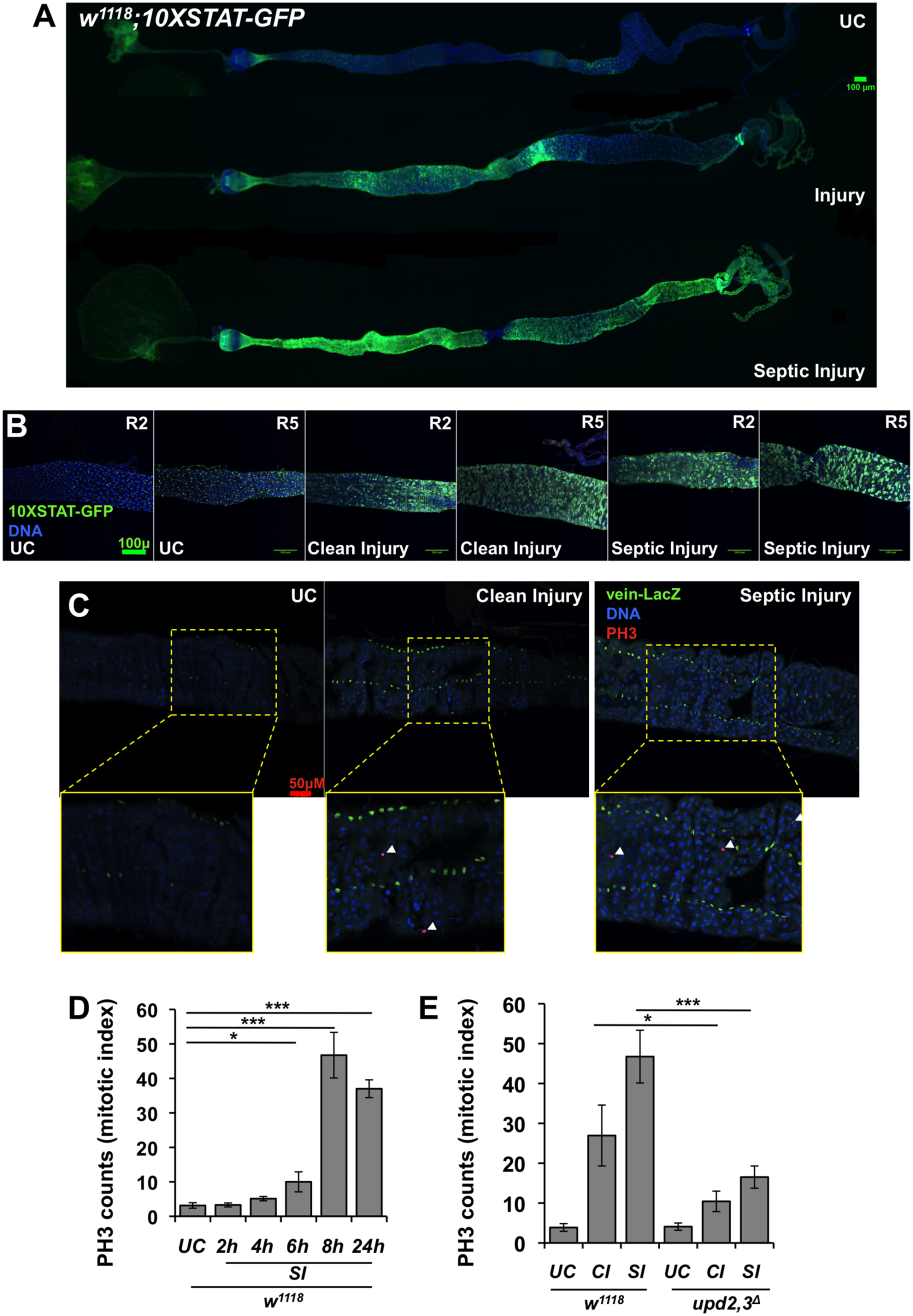
Clean and septic injury activates the JAK/STAT and EGFR pathways in the *Drosophila* intestine. (**A and B**) Immunostaining of the JAK/STAT reporter gene, *10XSTAT-GFP* with an anti-GFP antibody in the guts of flies upon injury and septic injury. (**A**) Representative images of entire guts are shown after 6 h post challenge. (**B**) Injury activates JAK/STAT in both visceral muscles and intestinal progenitor cells. Immunostaining revealed an expansion of the GFP signal in the guts upon clean or septic injury in wild-type flies. Shown are representative images of unchallenged (UC) flies or flies collected after a clean injury or a septic injury with *Ecc15*. Guts were observed 6 h post-challenge, R2 and R5 refer to region of the midgut. (**C**) Immunostaining against LacZ and PH3 of midgut of *vein-lacZ* flies reveals that the EGF-R ligand encoding gene, *vein* (nuclear signal), is induced upon clean and septic injury with *Ecc15* in the circular visceral muscles surrounding the intestine of female flies. Cuticle injury also increases the number of mitotic intestinal stem cells as revealed by an increased number of Phospho-Histone-3-positive cells (PH3+, white arrowheads) as seen clearer in the yellow boxed panels below that are zooms of the panels above. (**D**) The mitotic index of intestinal stem cell was quantified in wild-type (*w*^*1118*^) female flies by counting PH3 positive cells. (**E**) The mitotic index of *upd2*,*3*^*Δ*^ female flies as compared to the wild type (*w*^*1118*^) was determined in the midgut of unchallenged flies (UC) and in flies subjected to clean injury (CI) or septic injury (SI). All panels in this figure used guts from female adult flies. In (**D**) and (**E**) Mean values of at least three experiments (at least n = 10 gut for each experiment) ± SE are shown.

The precise function of the JAK/STAT pathway in the systemic immune response is still poorly defined. The pathway regulates a small subset of immune inducible genes, notably genes encoding stress peptides of the Turandot family, the opsonin Tep2 as well as Socs36E, a negative regulator of the JAK/STAT pathway [10–12]. Having shown that Upds are expressed in haemocytes and that some of them are required for resistance to infection, we next determined the impact of *upd2*,*3*^*Δ*^ deficiencies on JAK/STAT transcriptional activity. As expected, we observed a reduced expression of the JAK/STAT target genes *Socs36E*, *TotA* and *TotM* in *upd2*^*Δ*^, *upd3*^*Δ*^ and *upd2*,*3*^*Δ*^ deficient flies upon septic injury (Fig 3A, 3C and 3E). Next we wanted to explore where the JAK/STAT pathway was activated in wild-type flies upon septic injury, by monitoring the expression of *TotM* and *Socs36E* in three immune compartments: the gut, the carcass (comprising of the fat body) and haemocytes. Surprisingly, we observed that *Socs36E* was strongly induced in the gut and to a lower extent in the carcass upon septic injury (Fig 3B). *TotA* and *TotM* were induced mostly in the fat body, consistent with a previous study on *Drosophila* larvae [15] (Fig 3D and 3F). To further characterize JAK/STAT pathway activity *in vivo*, we used flies carrying the reporter gene *10XSTAT92E-eGFP*, which drives GFP expression under the control of ten STAT92E binding sites [29]. Since the same *10XSTAT92E-eGFP* transgene is induced in larval fat body upon septic injury (S2A Fig) and since a previous study has already shown that the transcription factor STAT is activated in the fat body of adult flies after septic injury [15], we would have expected the same in adults. However, we cannot exclude that a specific feature of the adult fat body hinders an accurate observation of GFP activity in this tissue. Interestingly, both clean and septic injury induces a significant expression of *10XSTAT92E-eGFP* in the gut (Fig 4A).

Altogether, our data indicate that Upd2 and Upd3 contribute to the activation of the JAK/STAT pathway following septic injury, and reveal that the gut is one of the main sites of JAK/STAT activation upon septic injury in adults.

### Body injury stimulates stem cell proliferation and immune response in the intestine

The JAK/STAT pathway has two main functions in the intestine. The first one is to promote epithelium renewal by stimulating the activity of intestinal stem cells and their differentiation into new enterocytes to rebuild the gut. The JAK/STAT pathway is activated through the release of Upd2 and Upd3 by stressed or damaged enterocytes, establishing a compensatory homeostatic loop [23, 30, 31]. Interestingly, the JAK/STAT pathway is also activated in the visceral muscles that surround the epithelium, regulating the production of the epidermal growth factor Vein, which stimulates the EGF-Receptor pathway in intestinal stem cells [30, 32]. The second function of the JAK/STAT pathway is to regulate a subset of peptides in the anterior part of the gut, with homology to the antimicrobial peptide Drosomycin: Drsl2, Drsl3 and Drl4. The expression of these Drosomycin-like peptides also depends on the JAK/STAT ligands Upd2 and Upd3, which are released by the intestinal epithelium following damage associated with oral bacterial infection [22]. Our observation that clean and septic injury induces STAT activity in the gut suggests that a body wound can remotely stimulate stem cell proliferation and an anti-microbial response in the intestine.

To investigate this, we further analyzed the site of *10XSTAT92E-eGFP* expression in the midgut of challenged adult flies. We noticed that the STAT reporter gene is induced both in the epithelium and in the visceral muscle that surrounds the epithelium (Figs 4A, 4B and S2B). In the epithelium, *10XSTAT92E-eGFP* signals are restricted to progenitor cells (stem cells and enteroblasts) in unchallenged flies, but the signal expands to enteroblasts and enterocytes after wounding and septic injury (Fig 4B). An expansion of *10XSTAT92E-eGFP* is suggestive of an increased epithelium renewal rate and is known to occur when flies are fed with infectious bacteria or corrosive agents, two conditions that stimulate stem cell activity [33, 34]. Supporting the hypothesis of increased epithelium turnover, the gene encoding the EGF ligand Vein is induced in the visceral muscles surrounding the gut following wounding and septic injury (Figs 4C and S4A). Intestinal epithelium renewal can easily be monitored in *Drosophila* by counting the number of dividing stem cells along the midgut using an anti-phosphohistone H3 (anti-PH3) antibody. Guts derived from flies collected after clean and septic injury exhibit an increased level of PH3 counts (Fig 4C–4E). Importantly, the midgut PH3 counts were strongly reduced, but not abolished, in *upd2*,*3*^*Δ*^ flies upon both clean and septic injury (Fig 4E for females and S3A Fig for males). We next investigated whether body injury can trigger the induction of Drosomycin-like peptides in the gut. *Drosomycin-like 3* (*Drsl3*) is indeed induced in the anterior part of the gut 6 h after septic injury (S4B Fig). In all these experiments we noticed that septic injury triggers a stronger response than a clean injury, indicating that the presence of bacteria increases the activation of JAK/STAT in the gut.

Taken together, our study uncovers a signaling pathway linking wound healing in the thorax to activation of stem cell activity and *Drosomycin-like* expression in the intestine. It also implies that Upds are required as a link between the wound and intestinal stem cell proliferation.

### The JAK/STAT activity in the gut is induced by the release of Upds from haemocytes

The results above show that JAK/STAT activity can be induced in the gut upon wounding and that this activation requires Upd2 and Upd3. However, they do not inform us in which tissue Upds are expressed. The increased stem cell proliferation and Drs-like induction upon body injury could be a secondary consequence of damage to the gut, which is known to produce unpaired ligands. This hypothesis is supported by a recent paper by Takeishi and colleagues, which showed that wounding induces apoptosis in the gut, which in turn activates the stem cell division pathway [21]. The authors postulated the existence of a ‘lethal’ factor that is released in the haemolymph upon wounding, which is responsible for intestinal damage. This prompted us to investigate whether clean or septic injury induces cell death in the gut that could explain the increase of STAT activity and epithelium renewal we observed. For this, we monitored apoptosis in the intestine of flies 6 h septic injury and 16 h after oral infection using an anti-caspase 3 staining and inspect caspase activation by monitoring the cleavage of DCP-1. We did not observe any increase of caspase 3 signal or cleavage of DCP-1 in flies following septic injury (S5A and S5B Fig). As expected, we observed a marked increase of apoptosis in the intestine of flies collected 16 h after oral infection with *Ecc15*, confirming the validity of the two apoptotic assays [30]. In light of these findings, we hypothesized that it might be rather the release of Upd ligands from circulating haemocytes that contributes to intestinal regeneration.

**Fig 5:**
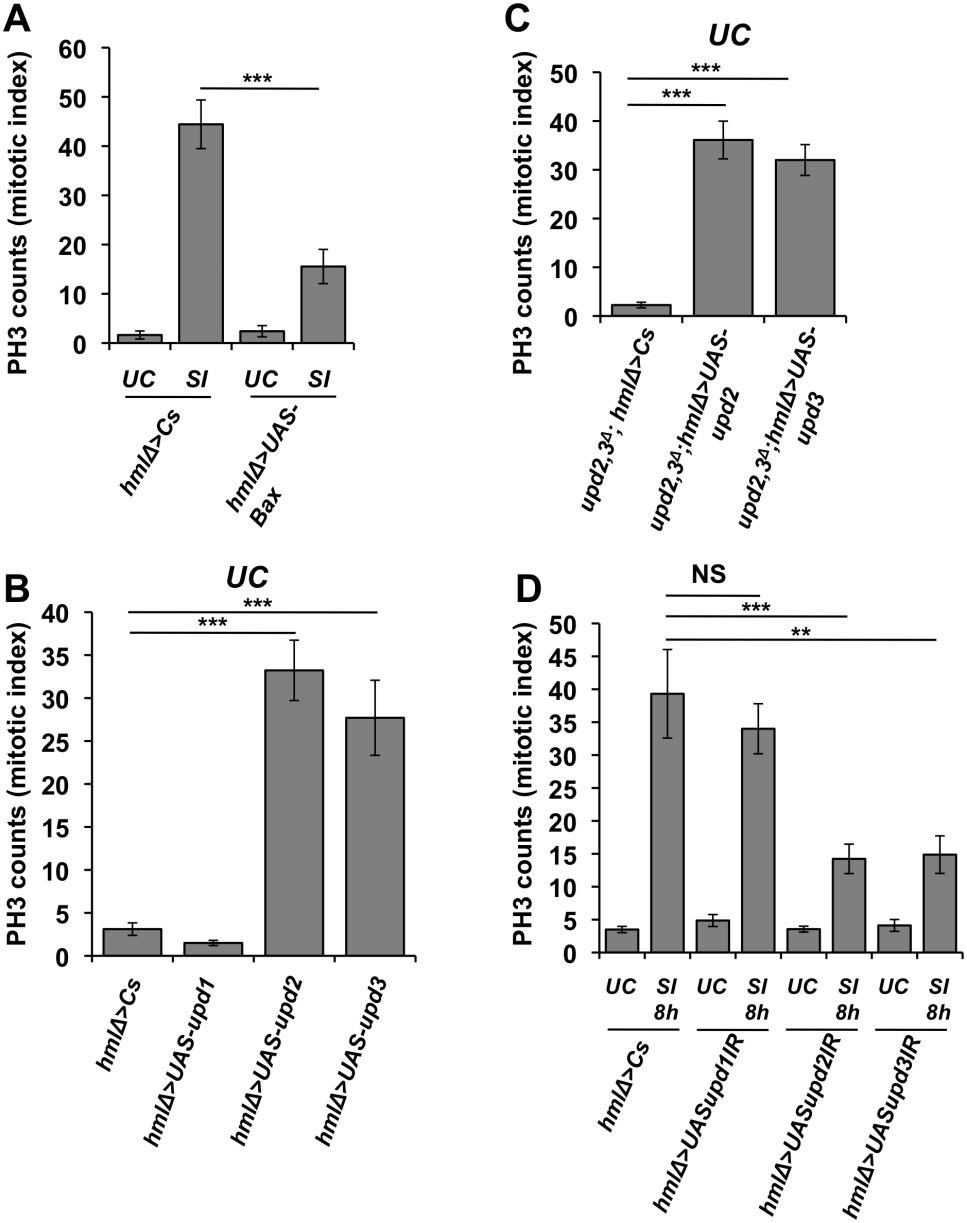
Haemocyte derived Upd ligands are necessary for proper intestinal stem-cell proliferation upon septic injury. (**A**) The midgut mitotic index of ‘hemoless’ female flies (*hmlΔGAL4* > *UAS*-*Bax*) confirms reduced proliferation as compared to the wild-type (*w*^*1118*^) upon septic injury with *Ecc15*. **(B)** The ectopic expression of *upd2* and *upd3* ligands but not *upd1* in adult fly haemocytes is sufficient to stimulate intestinal stem cell proliferation in absence of challenge as revealed by PH3 immunostaining. (**C**) The ectopic expression of *upd2* and *upd3* ligands from haemocytes in *upd2*,*3*^*Δ*^ adult female stimulates intestinal stem cell proliferation in absence of infection. (**D**) The midgut mitotic index of female flies with reduced haemocyte expression of *upd1*, *upd2* or *upd3* (*hmlΔGAL4/+*; *UAS*-*upd2-IR/+* or *hmlΔGAL4/+*; *UAS*-*upd3-IR/+*) revealed reduced intestinal stem cell proliferation as compared to controls. PH3 count in the midgut was determined by counting PH3 positive cells 8 h post septic infection with *Ecc15*. In (**A**-**D**) Mean values of at least three experiments (at least n = 10 gut for each experiment) ± SE are shown.

To test this notion, we monitored stem cell activity upon septic injury in flies lacking haemocytes due to the *hmlΔGal4* driven expression of Bax in plasmatocytes. Flies lacking haemocytes have a significantly lower intestinal mitotic index after septic injury as compared to their wild-type counterparts (Fig 5A). This strongly suggests that haemocytes do contribute to the signal that activates stem cell proliferation upon systemic wounding. We then tested whether over-expression of Upd ligands in haemocytes was sufficient to activate intestinal stem cell proliferation. Over-expression of *upd2* or *upd3* but not *upd1* using the haemocyte specific driver *hmlΔGal4*, stimulates an increased stem cell activity in the gut in the absence of wounding (Fig 5B). In contrast, we confirmed that over-expression of each of *upd2*, *upd3* and *upd1* (but a lower extent) in enterocytes (genotype: *MyoTS-Gal4*> *UAS-upd*) stimulates stem cell proliferation in agreement with previous studies [32]. The observation that the expression of *upd2* and *upd3* but not *upd1* in haemocytes can remotely activate stem cells is consistent with *in vivo* and *in vitro* data, showing that Upds have distinct diffusing properties and biological activities as suggested by previous studies [22, 35, 36]. We next tested whether the expression of Upds in haemocytes is sufficient to stimulate intestinal stem cell proliferation in the absence of any intestinal source of Upd2 and Upd3. Consistent with our hypothesis that haemocytes remotely activate intestinal stem cells, we observed that the over-expression of either *upd2* or *upd3* by haemocytes can stimulate intestinal stem cell proliferation in *upd2*,*3*^*Δ*^ flies (Fig 5C). In this experiment, we noticed that over-expression of Upd2, but not of Upd3, led to the accumulation of small nucleate cells in the gut of *upd2*,*3*^*Δ*^ flies, suggesting a differentiation defect (S4C Fig). This indicates that while hemolymphatic production of Upd2 or Upd3 can trigger gut stem cell proliferation, only Upd3 ensured proper epithelium renewal with differentiation of the proliferating stem cells, pointing to a specific requirement of *Upd2* to ensure proper enterocyte differentiation. Finally, to confirm that Upds derived from haemocytes play a role in septic injury induced intestinal stem cell proliferation, we used an *in vivo* RNAi approach to silence *upd1*, *upd2* or *upd3* in haemocytes using the *hmlΔGal4* driver, and monitored stem cell activity after septic injury. Fig 5D shows that reduction of *upd2* or *upd3* in haemocytes (but not *upd1*) resulted in decreased PH3 counts in the gut 8 h after a septic injury, compared to wild-type. We conclude that production of Upd2 and Upd3 by haemocytes upon systemic wounding is necessary for JAK/STAT activation in the gut. This uncovers a new mechanism where haemocytes can remotely activate intestinal stem cell activity.

### The JNK pathway regulates *upd3* expression upon septic injury in haemocytes

**Fig 6:**
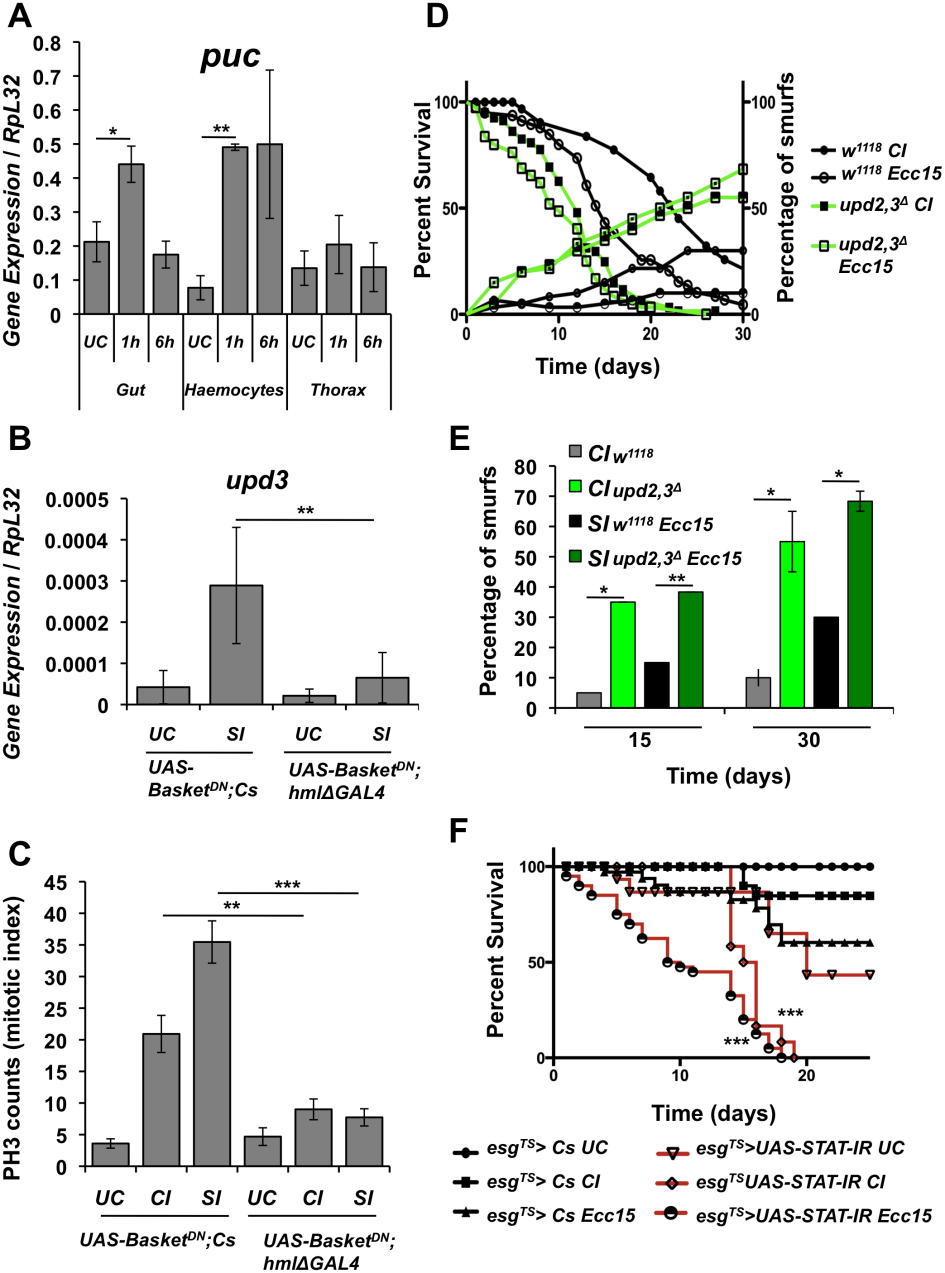
The JNK pathway regulates *upd3* expression in haemocytes. (**A**) The expression of *puckered* (*puc*), a readout of the JNK pathway, was monitored by RT-qPCR in haemocytes, fat body and gut of flies collected at 1 h and 6 h post-septic injury. (**B**) Reduction of JNK signaling in haemocytes by expressing a dominant-negative version of the JNK kinase Basket leads to a concomitant decrease in *upd3* expression in haemocytes collected 2 h after septic injury. (**C**) Female flies with reduced JNK signaling in haemocytes through the expression of dominant-negative form of the JNK Basket, display a reduced mitotic index in the midgut compared to the wild-type flies 8h after clean injury or septic injury. Mean values of at least three experiments (with at least 10 gut for each experiment) ± SE are shown. (**D**) Intestinal barrier function assay and survival curves of *w*^*1118*^ and *upd2*,*3*^*Δ*^ flies maintained on medium containing blue dye. The right Y-axis curve represents the cumulative proportion of Smurfs in the population. (**E**) Proportions Smurfs in wild-type (*w*^*1118*^) male flies and *upd2*,*3*^*Δ*^ male flies from day 10 and day 30 of adulthood. *upd2*,*3*^*Δ*^ flies display a higher proportion of Smurf phenotype after clean injury (CI) and septic injury (SI) as compared to their wild-type counterparts. n = 60 flies/condition. **: p< 0.01, *: p< 0.05 as determined by Student’s t-test. (**F**) Knockdown of JAK/STAT signaling specifically in intestinal progenitors using the *escargot*^*TS*^ driver leads to a higher mortality rate in flies subjected to either clean injury or septic injury. n = 60 flies per genotype that is pooled from three independent experiments. Log-rank test comparing *esg*^*TS*^*> UAS-STAT-IR* Clean Injury and *esg*^*TS*^ *> UAS-STAT-IR* Septic Injury to *esg*^*TS*^ *> Cs* Clean Injury and *esg*^*TS*^ *> Cs* Septic Injury respectively: *** = p, 0.001. RT-qPCR and survival analysis were done on adult male flies while PH3 counts were done with adult female flies.

Previous studies have established a link between JNK activity and the expression of Upds in the intestine [25, 31, 37]. We therefore investigated a possible role of the JNK pathway in the regulation of Upds in haemocytes upon septic injury. Interestingly, RT-qPCR analysis revealed that the JNK pathway is activated in both haemocytes and gut upon septic injury, as illustrated by the induction of *puckered*, a reporter gene of JNK pathway activity [38] (Fig 6A). We then monitored *upd2* and *upd3* expression and intestinal mitotic activity in flies expressing a dominant-negative form of the *Drosophila* JNK Basket in haemocytes [39]. Inactivation of JNK signaling reduces both haemocyte expression of *upd3* and intestinal PH3 counts upon septic injury, revealing that the stress-pathway JNK regulates *upd* gene expression in haemocytes (Fig 6B and 6C).

### Inhibition of intestinal epithelium renewal increases susceptibility to septic injury

Many studies have shown that dysfunction of the *Drosophila* intestine impacts both aging and resistance to stress in flies [40–42]. Along this line, Takeishi *et al*. have shown that a viable mutation in the caspase activator Apaf-1/dark, *dpf-1*^*K1*^, which leads to defects in gut epithelial renewal, results in lethality to wounding [21]. Based on these and our results, we hypothesized that the susceptibility of *upd2*^*Δ*^, *upd3*^*Δ*^ and *upd2*,*3*^*Δ*^ mutants to systemic wounding could be linked to an impairment in gut epithelium renewal. Recently, a method named the ‘Smurf assay’ has been developed to probe gut integrity. In this assay, flies are fed on food colored with a non-toxic water-soluble dye, and gut integrity is estimated by measuring the diffusion of the blue dye from the gut lumen to the hemocoel [41, 42]. ‘Smurf’ flies, which show systemic diffusion of blue dye due to intestinal barrier dysfunction, exhibit a lower life expectancy. We used this assay to assess gut barrier integrity of *upd2*,*3*^*Δ*^ flies. *Upd2*,*3*^*Δ*^ flies display a higher proportion of ‘Smurf’ flies over time upon wounding and septic injury (Fig 6D and 6E). The kinetics of ‘Smurf’ occurrence correlates with the survival to clean and septic injury. A higher proportion of ‘Smurf’ flies over time was also observed in *hmlΔGal4 > UAS-Bax* haemocyte-less flies compared to wild-type (S3B Fig). Hence, an accelerated loss of gut integrity in *upd2*,*3*^*Δ*^ flies is likely underlying their enhanced susceptibility to injury. To further test this hypothesis, we knocked down the JAK/STAT pathway specifically in the gut progenitor cells using *esg*^*ts*^*GAL4*, and monitored survival to septic injury. In these experiments, a dominant-negative form of the receptor Domeless (*Domeless*^*DN*^) or a *STAT RNAi* construct (*STAT-IR)* were expressed in 3 day old adults using the thermo-sensitive driver *esg*^*ts*^*GAL4*. Flies with reduced JAK/STAT activity in progenitor cells failed to survive clean and septic injury (Figs 6F and S4D). Thus, our results identify intestinal epithelium renewal as a critical event for survival to septic injury.

### Activation of intestinal stem cells upon oral infection does not require haemocytes

**Fig 7:**
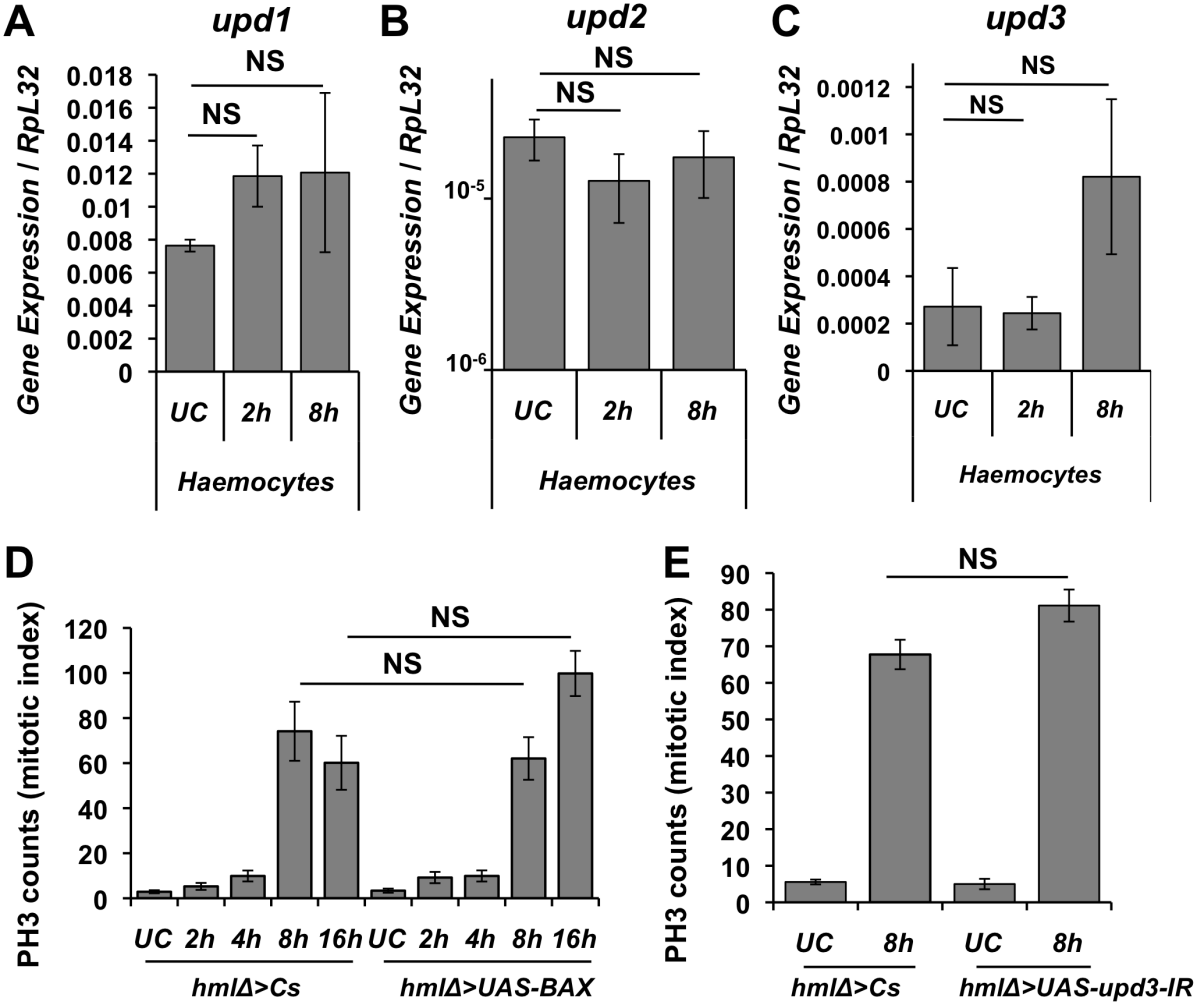
Haemocytes do not contribute to intestinal stem cell proliferation upon oral bacterial infection. (**A-C**) RT-qPCR experiments show that *upd1*, *upd2* and *upd3* expression is not significantly changed in haemocytes after oral infection with *Ecc15*. P values were determined by Mann-Whitney U test for **A**-**C.** P = 0.1 (2h and 8h vs UC) for *upd1*; P = 0.4 (2h vs UC) and 0.7 (8h vs UC) for *upd2*; P = 0.7 (2 h and 8 h vs UC) for *upd3*. (**D and E**) Kinetic analysis of the midgut mitotic index of female flies ablated for haemocytes (D: *hmlΔGAL4* > *UAS-BAX*) or with reduced *upd3* haemocyte expression (E: *hmlΔGAL4* > *UAS-upd3-IR*). Ablation of haemocytes does not affect intestinal stem cell proliferation as determined by counting PH3+ cells 8 h and 16 h after oral infection with *Ecc15*. RT-qPCR was done on male flies while PH3 counts were done on female flies.

Our study is not the first to identify a role for haemocytes in intestinal epithelium renewal. Ayyaz *et al*. (2015) have recently shown that adult haemocytes are required for proper stem cell proliferation upon ingestion of paraquat and oral bacterial infection via the release of the Dpp ligand [43]. They also reported some haemocytes are recruited to the intestine upon stresses such as oral bacterial infection. This study does not preclude that haemocytes could also be a source of Upds in those conditions. To test this, we first investigated whether haemocytes respond to oral bacterial infection by expressing *upds*. *Upd3* was slightly induced in haemocytes upon oral infection with *Ecc15*, but the level of increase did not reach statistical significance (Fig 7A–7C). The relative gene expression level of *upd3* over *RpL32* indicates that *upd3* expression after oral infection in haemocytes was much weaker as compared to septic injury with *Ecc15* (Fig 2C). This indicates that haemocytes are more reactive to systemic infection than oral infection. We next monitored intestinal stem cell proliferation in the midgut of flies expressing *upd3* RNAi in haemocytes, or of flies lacking nearly all haemocytes due to the expression of pro-apoptotic genes *Bax* (see above) or *hid*. Neither the absence of plasmatocytes, nor the silencing of *upd3* in haemocytes affected the gut mitotic index in response to oral bacterial infection (Figs 7D, 7E and S6B). Use of an *HmlΔ-Gal4*, *UAS-GFP* reporter strain reveals an aggregation of haemocytes close to the midgut at the level of the loop (S6A Fig, as described by Ayyaz *et al*. [43]). Nevertheless, we did not observe any obvious change in haemocytes number around the gut upon septic injury or oral bacterial infection with *Ecc15*. We thus conclude that the intestinal repair response to oral bacterial infection can occur in absence of haemocytes.

## Discussion

Although it is recognized that three signaling pathways in *Drosophila*, Toll, Imd, and JAK/STAT, mediate the bulk of the transcriptional response to septic injury, functional studies on the JAK/STAT pathway have been hampered by the fact that loss-of-function mutations in genes encoding components of this pathway cause severe developmental defects. In this study, we have used viable mutations in *upd2* and *upd3* and tissue-specific inactivation of JAK/STAT components to assess their functions in systemic immunity. We demonstrate that Upd-JAK/STAT signaling is required to resist wounding and septic injury in adults. The *upd2*,*3*^*Δ*^ deficient flies show a mild but clear susceptibility ten days after wounding. This is consistent with the common view that this pathway responds to damage, while Toll and Imd pathways are more responsive to microbial infection. Thus, the Upd-JAK/STAT axis should be seen as an integral part of the systemic wound response.

**Fig 8:**
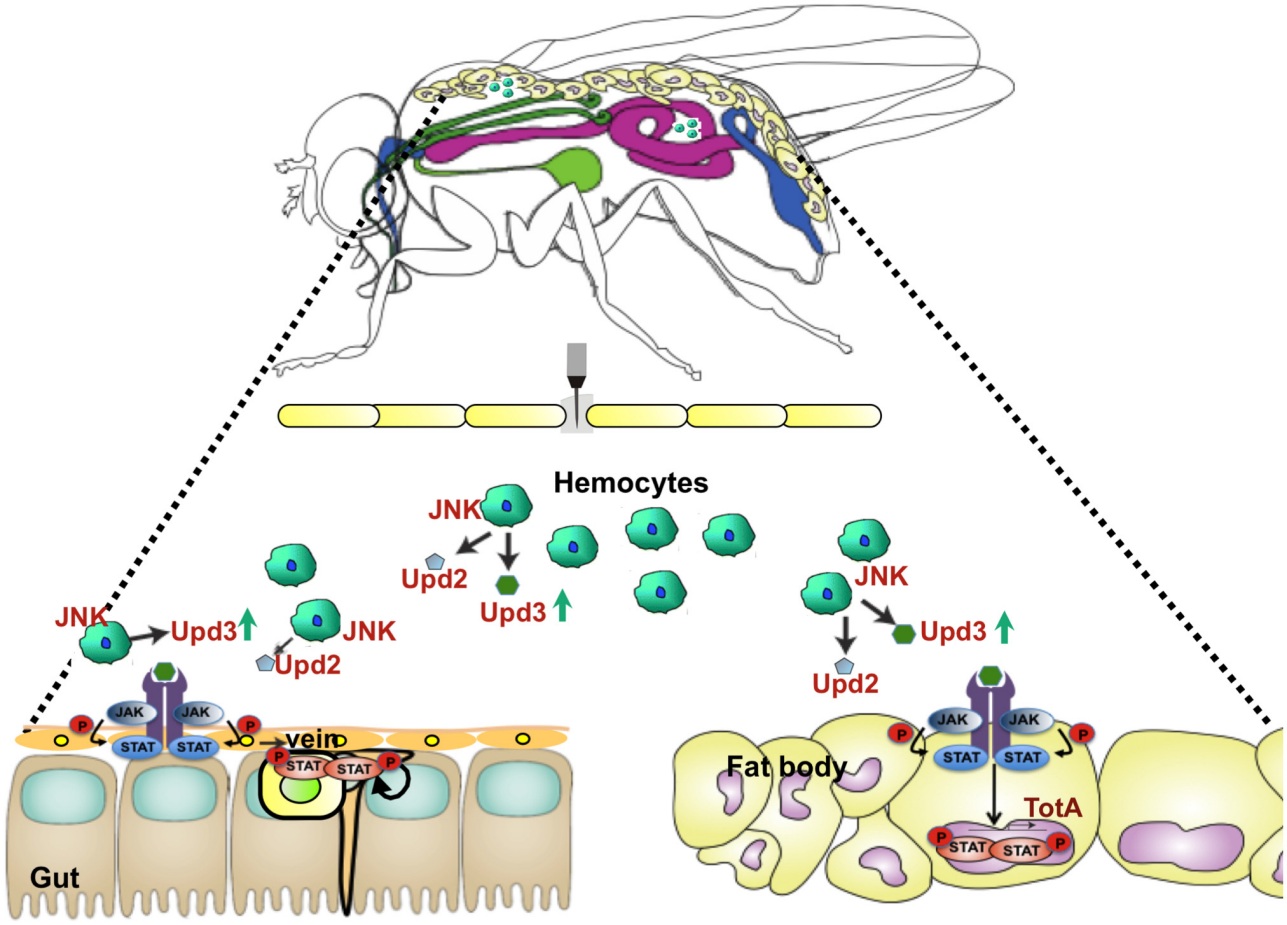
Model illustrating the central role of haemocytes and Upds in the systemic wound response. Upon injury or septic injury, haemocytes release Upd3 into the haemolymph. Upd3 with Upd2 then activates the JAK/STAT pathway in both progenitor cells and in the surrounding visceral muscles to stimulate intestinal stem cell proliferation and differentiation. The modulation of the gut epithelium renewal by wounding and septic injury then promotes survival of flies. It is likely that septic injury damages the intestine, and that haemocytes provides an anticipatory mechanism to repair the gut.

Agaisse *et al*. 2003 initially reported that JAK/STAT activation in the larval fat body is mediated by a ligand, Upd3, which is secreted by haemocytes [15]. Our data confirm and extend this finding, showing that the production of Upd3 by haemocytes activates JAK/STAT signaling in the adult fat body (See model Fig 8). It also shows that haemocytes produce two other *upds*, Upd1 and Upd2, whose expression is less sensitive to septic injury. However, the observation that haemocytes contribute to intestinal epithelium renewal in response to septic injury was unexpected. Takeishi *et al*. (2013) were the first to establish a link between systemic wounding and intestinal homeostasis. They showed that body wounds remotely control caspase activity in enterocytes, which in turn activates the intestinal regeneration pathway in the gut [21]. However, the authors of this paper did not identify the link between body injury and the induction of apoptosis in the gut, they postulated the existence of a haemolymph ‘lethal’ factor that contributes to intestinal damage. Our study demonstrates that the wounding of the cuticle can activate the production of Upds by haemocytes, and that these secreted ligands are sufficient to induce stem cell proliferation in the intestine. Our observation that Upds released by haemocytes can remotely stimulate stem cell activity indicates that epithelium renewal is influenced by insults from both the luminal (microbial infection, ingested toxins) and the hemolymphatic compartments (septic injury). Along the same line, it is well known that gut dysfunction and subsequent loss of appetite in humans commonly occurs following surgery or major injury [44], although the molecular mechanisms of this process are poorly understood.

The reason why septic injury stimulates intestinal stem cells remains enigmatic. Takeishi *et al*. (2013) and our study suggest that the intestine is an organ sensitive to systemic wounding. In agreement with this notion, the ‘Smurf’ assay reveals an increased intestinal permeability in *upd2*,*3*^*Δ*^ flies upon septic injury. Stem cell proliferation is still activated, albeit at a much lower rate, in *upd2*,*3*^*Δ*^ and in haemocyte-depleted flies. This suggests that additional factors beyond Upds, can stimulate intestinal stem cell proliferation. A first hypothesis would be that septic injury with a needle directly damages the intestine [21]. We do not favor this notion because the intestine did not appear damaged upon septic injury as assessed by phalloidin staining of the entire gut (S2B1 and S2B4 Fig) and did not show an increase of apoptosis at early time points (S5A and S5B Fig). A second hypothesis is that wounding of the cuticle indirectly alters gut integrity. For instance, reactive oxygen species (ROS) induced during the systemic wounding reaction could damage the intestinal basement membrane. Thus, the production of Upds by haemocytes could be a way to stimulate intestinal repair as an anticipatory tissue repair mechanism.

The role of the JAK-STAT pathway in the systemic wound response is not limited to the fat body and the gut. The JAK/STAT also plays a major role in larval haemocytes where it contributes to haemocyte recruitment or the differentiation into lamellocytes [19, 20]. A recent study has shown that infestation by the parasitoid wasp *Leptopilina boulardi* triggers the release of Upd2 and Upd3 from haemocytes, which in turn activate the JAK/STAT pathway in the larval somatic muscles [45]. Collectively, haemocyte-derived Upds stimulate a broad array of responses in multiple tissues, and future studies should decipher how each organ contributes to the systemic wound response. Blocking the JAK/STAT pathway in the gut makes flies susceptible to a simple injury, suggesting that proper gut function is essential for recovery from systemic injury. Therefore, it cannot be excluded that several roles initially attributed to the JAK/STAT pathway are linked to its ability to turn on epithelium renewal in the intestine. For instance, the involvement of the JAK/STAT pathway in the resistance to *Drosophila* C Virus and cricket paralysis viruses in *Drosophila* or its implication in the defense against *Plasmodium* in *Anopheles* [46–49] could be linked to a defect in epithelium renewal.

Our study reveals that haemocytes are dispensable to epithelium renewal upon oral infection with *Ecc15*. Thus, haemocytes promote epithelium repair in response to a systemic threat but are less important in the defense against oral bacterial infection. Our results diverge from those of a recent study, which pointed to a contribution of adult haemocytes to stem cell proliferation, via their release of the Dpp ligand upon ingestion of paraquat or oral bacterial infection [43]. Consistent with the work of Ayyaz *et al*., (2015), we did observe aggregation of haemocytes close to the midgut at the level of the loop. Nevertheless, we did not observe any obvious recruitment of haemocytes to the gut upon septic injury or oral bacterial infection with *Ecc15*. The continuous presence of haemocytes in close proximity to the midgut could allow a local enrichment of the diffusible JAK/STAT ligands upon septic injury [43]. The presence of haemocytes in close proximity to many tissues including gut and imaginal discs [20, 50] underlines their function in tissue homeostasis as local tissue repairers.

Our study delineates a pathway in which damages associated with injury induce the release of Upd3 by haemocytes to activate JAK/STAT in the intestine both in the visceral muscles and stem cells (Fig 8). We further show that the JNK pathway regulates the expression of *upd3* as previously described for the gut [25, 31, 41]. This is in line with a former study describing a role for the JNK pathway component Mekk1 in the expression of the JAK/STAT target genes of the *Turandot* family, although the tissue where Mekk1 is required had not been identified [18]. Another recent study already implicated the JNK pathway for the regulation of Upd3 production in haemocytes in response to the ingestion of a high-lipid diet [51]. The observation that septic injury triggers *upd3* expression in haemocytes more effectively than clean injury suggests that the Imd and Toll pathways, which sense microbial determinants, could promote the induction of *upds* in haemocytes possibly through a convergence on the JNK pathway.

Systemic wounding induces a complex series of events that includes the entry of oxygen, rupture of basement membranes, ROS burst, production of melanin, as well as aggregation of clotting factors and haemocytes around the wound [52, 53]. Wounding also leads to the release of cytoplasmic material from dead cells such as cytoplasmic actin, which functions as a DAMP in mammals [54]. A major challenge is now to identify the underlying mechanism that senses damage and activates the pathway. Previous studies have shown that H_2_O_2_ is a primary local signal responsible for the homing of haemocytes to the wound site in embryos [28, 55]. An attractive hypothesis is that ROS may be the signal activating the JNK pathway in the haemocytes close to the wound site. Alternatively, it cannot be excluded that haemocytes harbor a receptor capable of recognizing a component normally restricted to the cytoplasm of living cells and unable to cross the basement membrane. This would explain why they are activated only by the concomitant destruction of both the basement membrane and the underlying epithelium. Our study emphasizes the central role of haemocytes via the production of Upds in an integrated systemic wound response. Future studies should now address how damage is sensed by haemocytes and how the JAK/STAT pathway targets genes contributes to repair programs and host survival.

## Materials and Methods

### *Drosophila* stocks and rearing

*Canton*^*S*^ (*Can*^*S*^) and *w*^*1118*^ flies were used as wild-type controls. The following fly lines were used in this study are *upd2*^*Δ*^, *upd3*^*Δ*^ and *upd2*,*3*^*Δ*^ [22]; *w;UAS-upd1-IR* (VDRC # 3282), *w;UAS-upd2-IR* (NIG # 5988 R1-3) *w;UAS-upd3-IR* [15], *hml*Δ*Gal4*,*UAS-GFP* [26], *w;;UAS-Dome*^*DN*^ [56], *yw;vein-lacZ(P1719)*,*FRT82B/TM6B* [32], *w*, *UAS-upd1* [36], *w*, *UAS-upd2* [33], *w*, *UAS-upd3* [31], *w;Gal80*^*TS*^*/CyO;howGal4*^*24B*^ [30], *Myo1AGal4;Gal80*^*TS*^ [25], *w;10XSTAT-GFP* [28], *UAS-Bsk*^*DN*^ [39], *UAS-hid* [57]. The F1 progeny of *hmlΔGal4*,*UASGFP* females crossed with *UAS-Bax* males were used to produce haemocyte defective flies, as described in [58]. RNAi flies carrying a GAL4 construct and UAS-IR construct were raised at 18°C during their larval and pupal development, and then shifted to 29°C for 8 days to activate the UAS-IR. All stocks were reared on standard fly medium comprising of 6% cornmeal, 6% yeast, 0.62% agar, 0.1% fruit juice, that was supplemented with 10.6g/L moldex and 4.9ml/L propionic acid and were maintained at 25°C on a 12 h light/ 12 h dark-cycle. Unless otherwise stated, experiments are done in male flies.

### Bacterial strains and analysis of bacterial counts

*Erwinia carotovora carotovora 15 (Ecc15)* is a Gram-negative bacterium described in [59], while *Enterococcus faecalis* (*E*. *faecalis*) and *Microccus luteus (M*. *luteus)* are both Gram-positive bacteria [14]. *Ecc15* and *M*. *luteus* were cultured overnight in LB medium at 29°C, and *E*. *faecalis* was cultured at 25°C.

For bacterial counts, *w*^*1118*^ and *upd2*,*3*^*Δ*^ male flies were infected with *E*. *faecalis*, and the number of bacteria was determined as follows at 10 h and 50 h post-infection [60]. Flies were surface sterilized in 95% ethanol for 1 min, and then 5 flies were homogenized using a Precellys 24 instrument (Bertin Technologies, France), and then five 1/10 serial dilutions were made and plated on LB culture medium by spotting the serial in duplicates and incubated overnight at 25°C. Colonies were counted from spots containing more than 10 colonies. The experiment was repeated three times.

### Infection and survival experiments

For septic injury and natural infection experiments, *Drosophila* 3–4 days old adults were used. For septic injury, male flies were pricked in the thorax with a needle dipped into a concentrated culture of *Ecc15* (OD_600_ ~200), *M*. *luteus* (OD_600_ ~200) and *E*. *faecalis* (OD_600_ ~10). After septic injury, flies were incubated at 29°C for *Ecc15* and *M*. *luteus* infection and at 25°C for *E*. *faecalis* infection. For oral infection, batches of 20 adult female flies were starved for 2 h at 29°C in an empty vial before being transferred to a fly vial with an infection solution. The infection solution consisted of an equal volume of 100X concentrated pellet from an overnight culture of *Ecc15* (OD_600_ = 200) with a solution of 5% sucrose (1:1) which was deposited on a filter disk that completely covered the surface of standard fly medium [59]. Flies were incubated for one day at 29°C on the contaminated filter, after which they were transferred to fresh vials containing standard medium without living yeast. In survival experiments, flies were maintained on medium without fresh yeast following infection and survivors were counted daily.

### RT-qPCR

10–15 adult male flies or 20 dissected guts (including crop, midgut and hindgut but without Malpighian tubules), or 15 carcasses (that comprise mostly fat body) were collected in 500 μl of Trizol (Invitrogen). For collecting haemocytes, 20 individuals were placed on a 30 μM filter of an empty Mobicol spin column (MOBITEC), then covered with glass beads and centrifuged for 20 minutes at 4°C, 10’000 r.p.m. This was done twice for a total of 40 adult male flies, and then the haemolymph recovered was pooled and collected in 300 μl of Trizol. Total RNA was extracted according to the manufacturer’s instructions. Quality of the RNA was determined using a NanoDrop ND-1000 spectrophotometer. The purified RNA was then treated with DNAse (Ambion) according to the manufacturer’s instructions. The quantity of RNA was determined using the NanoDrop and then 1 μg of RNA was used to generate cDNA using SuperScript II (Invitrogen, Carlsbad, California, United States). RT-qPCR was performed using dsDNA dye SYBR Green I (Roche Diagnostics, Basel, Switzerland). Expression values were normalized to *RpL32*. Primer sequences used are provided in S1 Table.

### Imaging and immunohistochemistry

For immunofluorescence, guts from 3 to 5 day old females were dissected in 1X PBS, fixed for 20 minutes in PBS, 0.1% Tween 20 (PBT), and 4% paraformaldehyde; then stained with primary antibody [1/1000 mouse anti-GFP (Roche); 1/500 rabbit anti-PH3 (Upstate/Millipore)]; 1/200 rabbit anti-Caspase 3 activity (ThermoFischer); 1/200 rabbit anti-cleaved DCP1 (Cell Signaling Technology) in PBT + 2% BSA]. Secondary staining was performed with Alexa594 anti-rabbit and Alexa488 anti-mouse antibodies (Invitrogen). Visceral muscles were stained using 1/500 Phalloidin-Rhodamine or 1/500 Phalloidin-FITC (Life Technologies). DNA was stained with 1/15000 dilution of 4’,6- diamidino-2-phenylindole DAPI (Sigma). The stained gut tissue was mounted in the antifading agent Citifluor AF1 (Citifluor Ltd.). The mitotic index was determined by counting the number of PH3 positive cells along the midgut with Axioplot imager (Zeiss). For determination of PH3 counts, at least 10 guts were counted per condition in each experiment and data was pooled from 3 independent experiments. The mean number of mitoses per midgut and S.E.M. are shown for each genotype or treatment in the graphs.

### Statistics

Each experiment was repeated independently a minimum of three times (unless otherwise indicated), error bars represent the standard error of the mean of replicate experiments (unless otherwise indicated). Statistical significance was determined using Student’s t test, Mann-Whitney U test or log–rank test on GraphPad Prism, and P values of <0.05 = *, <0.01 = ** and <0.001 = *** for the t test and Mann-Whitney test while P <0.0001 = *** for log-rank test were considered significant.

## Supporting Information

S1 Fig *Upd2*^*Δ*^, *upd3*^*Δ*^ and *upd2*,*3*^*Δ*^ flies have wild-type Toll and IMD pathway activity. (**A and B**) RT-qPCR analysis of *Diptericin* (*Dpt*) and *Drosomycin* (*Drs*) expression in adults either unchallenged (UC) or collected at 6 h and 24 h after septic injury with *Ecc15* (**A**) or *M*. *luteus* (**B**). Data are the mean of three repeats ± SE. The P value was determined NS (non-significant) using pair-wise comparison between each time-point of *upd2*^*Δ*^, *upd3*^*Δ*^ and *upd2*,*3*^*Δ*^ infected to *w*^*1118*^ infected using Student’s *t*-test. (**C**) Knockdown of both *upd2* and *upd3* in haemocytes using the *PxnGAL4* driver leads to a higher mortality rate in flies subjected to either clean injury or septic injury with *Ecc15*. Data is pooled from three independent experiments, n = 60. (**D**) ‘Hemoless’ flies (*hmlΔGAL4* > *UAS*-*BAX*) display an increased susceptibility to wounding as well as septic injury with *Ecc15*. Data is pooled from three independent experiments, n = 60. The log-rank test was used to determine statistical significance. P value < 0.001 = *** as determined by log–rank test for *hmlΔGAL4* > *UAS*-*BAX* CI and *Ecc15* SI as compared to *hmlΔGAL4* > *Cs* CI and *Ecc15* SI; P value < 0.001 = *** for *pxnGAL4 > UAS-upd2*,*3IR* CI and *Ecc15* SI compared to *pxnGAL4*> *Cs* CI and *Ecc15* SI. Male flies were used for experiments done in panels **A**-**D**.
(TIF)

S2 Fig Upd2 and Upd3 ligands contribute to JAK/STAT pathway in the fat body and the intestines upon injury. **(A)** A *10XSTAT-GFP* (green) reporter allele was used to monitor JAK/STAT activity in a living third-instar larva. Live-imaging of larvae at 6 h post-injury revealed an increase of GFP signal in the fat body and the gut. No increase in GFP signal was observed in the *upd2*,*3*^*Δ*^ larvae upon injury. Representative images from unchallenged (UC) flies or flies collected 6 h after clean injury or septic injury. The white arrowheads in wild-type clean injury and septic injury indicate GFP signal in the gut of larvae while white arrows indicate GFP in the fat body of the head. (**B1-B4**) Immunostaining on *10XSTAT-GFP* (green) reporter gene and visceral muscle using Rhodamine-phalloidin (red) in the intestine region R2 of adult female flies either unchallenged (UC) or collected 6 h after septic injury with *Ecc15*. Nuclei are stained using DAPI (blue) and co-staining of *10XSTAT-GFP* expression with the visceral muscle is seen in yellow.
(TIF)

S3 Fig Haemocytes contribute to gut integrity after septic injury. **(A)** The mitotic index of *upd2*,*3*^*Δ*^ male flies as compared to the *w*^*1118*^ in the midgut of unchallenged flies (UC) and in flies subjected to clean injury (CI) or septic injury (SI). Males show a reduced intestinal stem-cell proliferation as compared to females after injury and septic infection (Compare Figs 5A and S3A, [61]). **(B)** Smurf assay and survival curves for *hmlΔGAL4* > *UAS*-*Bax Ecc15* SI. The right Y-axis curve is the cumulative proportion of Smurfs in the population. *hmlΔGAL4* > *UAS*-*Bax* flies display higher proportion of Smurf phenotype after septic injury (SI) as compared to their wild-type counterparts. (**C**) Proportions Smurfs in *hmlΔGAL4* > *Cs* SI male flies and *hmlΔGAL4* > *UAS*-*Bax Ecc15* SI male flies at day 10 and day 20 after infection. 60 flies/condition were used, and **: p< 0.01, *: p< 0.05 as determined by Student’s t-test.
(TIF)

S4 Fig Septic injury induces the expression of *vein* and *Drsl3* in the intestine upon septic injury. **(A)** The expression of *vein* was measured 1 h and 6 h after septic injury. (**B**) RT-qPCR experiments show that the putative antimicrobial peptide gene *Drsl3* is induced in the intestine upon septic injury. (**C**) Over-expression of Upd2 in haemocytes in *upd2*,*3*^*Δ*^ flies(genotype: *upd2*,*3*^*Δ*^, *UAS-upd2/+; hmlΔGAL4/+)* leads to the accumulation of small nuclei cells in the adult intestine as revealed by DAPI staining, pointing to a defect in differentiation. (**D**) Reducing JAK/STAT signaling by over-expression of a dominant-negative form of the receptor Domeless (*UAS-Dome*^*DN*^) using the intestinal progenitor specific driver *escargot*^*TS*^, leads to an increased mortality in male flies subjected to either a clean injury or a septic injury. Data is pooled from four independent experiments. The log-rank test was used to determine statistical significance. P value < 0.001 = *** as determined by log–rank test for *esg*^*TS*^*-GAL4*; *UAS*-*Dome*^*DN*^ CI and *Ecc15* SI as compared to *esg*^*TS-*^*GAL4*; *Cs* CI and *Ecc15* SI.
(TIF)

S5 Fig Septic injury does not induce apoptosis in the intestine. (**A**-**B**) Immunostaining using antibodies directed against activated Caspase 3 (**A**) and cleaved DCP-1 (**B**) shows that oral infection with *Ecc15* but not septic injury induces an increase the level of apoptosis. Shown is the R2 region of female midgut from flies either unchallenged (UC) or collected 6 h after septic injury and 16 h after natural infection with *Ecc15*. Nuclei are stained using DAPI (blue) and co-staining of Caspase 3 in red.
(TIF)

S6 Fig Haemocytes are not required for stem cell proliferation upon natural infection. (**A**) Visualization of gut-associated haemocytes using *hmlΔGAL4>UAS-RFP flies*. Intestines were fixed with 4% paraformaldehyde and stained with FITC-phalloidin to label visceral muscle and DAPI to label nuclei. While the number of haemocytes attached to the loop of the midgut was variable, we did not observe any marked variation in haemocytes number upon septic injury. (**B**) ‘Hemoless’ female flies due to the overexpression of the pro-aptototic gene *hid* in hemocytes have wild-type level of intestinal stem cell proliferation 8 h after oral infection with *Ecc15* as determined by counting PH3+ cells. Genotype: *hmlΔGAL4* > *UAS*-*hid*.
(TIF)

S1 Table List of qPCR primer sequences used in this study. (DOCX)

## References

[1] Akira S, Uematsu S, Takeuchi O. Pathogen recognition and innate immunity. Cell. 2006;124:783–801. 16497588 10.1016/j.cell.2006.02.015

[2] Medzhitov R. Recognition of microorganisms and activation of the immune response. Nature. 2007;449:819–26. 17943118 10.1038/nature06246

[3] Gallucci S, Matzinger P. Danger signals: SOS to the immune system. Curr Opin Immunol. 2001;13:114–9. 11154927 10.1016/s0952-7915(00)00191-6

[4] Rock KL, Lai JJ, Kono H. Innate and adaptive immune responses to cell death. Immunol Rev. 2011;243:191–205. 10.1111/j.1600-065X.2011.01040.x 21884177 PMC3170128

[5] Stark GR, Darnell JE Jr. The JAK-STAT pathway at twenty. Immunity. 2012;36:503–14. 10.1016/j.immuni.2012.03.013 22520844 PMC3909993

[6] Myllymaki H, Ramet M. JAK/STAT pathway in Drosophila immunity. Scand J Immunol. 2014;79:377–85. 10.1111/sji.12170 24673174

[7] Binari R, Perrimon N. Stripe-specific regulation of pair-rule genes by hopscotch, a putative Jak family tyrosine kinase in Drosophila. *Genes Dev* 1994;8:300–12. 8314084 10.1101/gad.8.3.300

[8] Zeidler MP, Bausek N. The Drosophila JAK-STAT pathway. JAKSTAT. 2013;2:e25353 10.4161/jkst.25353 24069564 PMC3772116

[9] Harrison DA, McCoon PE, Binari R, et al Drosophila unpaired encodes a secreted protein that activates the JAK signaling pathway. Genes Dev. 1998;12:3252–63. 9784499 10.1101/gad.12.20.3252 PMC317220

[10] Barillas-Mury C, Han YS, Seeley D, et al Anopheles gambiae Ag-STAT, a new insect member of the STAT family, is activated in response to bacterial infection. EMBO J. 1999;18:959–67. 10022838 10.1093/emboj/18.4.959 PMC1171188

[11] Lamiable O, Kellenberger C, Kemp C, et al Cytokine Diedel and a viral homologue suppress the IMD pathway in Drosophila. Proc Natl Acad Sci U S A. 2016;113:698–703. 10.1073/pnas.1516122113 26739560 PMC4725508

[12] Lagueux M, Perrodou E, Levashina EA, et al Constitutive expression of a complement-like protein in toll and JAK gain-of-function mutants of Drosophila. Proc Natl Acad Sci U S A. 2000;97:11427–32. 11027343 10.1073/pnas.97.21.11427 PMC17216

[13] Boutros M, Agaisse H, Perrimon N. Sequential activation of signaling pathways during innate immune responses in Drosophila. Dev Cell. 2002;3:711–22. 12431377 10.1016/s1534-5807(02)00325-8

[14] Stec W, Vidal O, Zeidler MP. Drosophila SOCS36E negatively regulates JAK/STAT pathway signaling via two separable mechanisms. Mol Biol Cell. 2013;24:3000–9. 10.1091/mbc.E13-05-0275 23885117 PMC3771960

[15] Agaisse H, Petersen UM, Boutros M, et al Signaling role of hemocytes in Drosophila JAK/STAT-dependent response to septic injury. Dev Cell. 2003;5:441–50. 12967563 10.1016/s1534-5807(03)00244-2

[16] De Gregorio E, Spellman PT, Tzou P, et al The Toll and Imd pathways are the major regulators of the immune response in Drosophila. EMBO J. 2002;21:2568–79. 12032070 10.1093/emboj/21.11.2568 PMC126042

[17] Ekengren S, Hultmark D. A family of Turandot-related genes in the humoral stress response of Drosophila. Biochem Biophys Res Commun. 2001;284:998–1003. 11409894 10.1006/bbrc.2001.5067

[18] Brun S, Vidal S, Spellman P, et al The MAPKKK Mekk1 regulates the expression of Turandot stress genes in response to septic injury in Drosophila. Genes Cells. 2006;11:397–407. 16611243 10.1111/j.1365-2443.2006.00953.x

[19] Luo H, Hanratty WP, Dearolf CR. An amino acid substitution in the Drosophila hopTum-l Jak kinase causes leukemia-like hematopoietic defects. EMBO J. 1995;14: 1412–1420. 7729418 10.1002/j.1460-2075.1995.tb07127.x PMC398227

[20] Pastor-Pareja JC, Wu M, Xu T. An innate immune response of blood cells to tumors and tissue damage in Drosophila. Dis Model Mech. 2008;1:144–54. 10.1242/dmm.000950 19048077 PMC2562178

[21] Takeishi A, Kuranaga E, Tonoki A, et al Homeostatic epithelial renewal in the gut is required for dampening a fatal systemic wound response in Drosophila. Cell Rep. 2013;3:919–30. 10.1016/j.celrep.2013.02.022 23523355

[22] Osman D, Buchon N, Chakrabarti S, et al Autocrine and paracrine unpaired signaling regulate intestinal stem cell maintenance and division. J Cell Sci. 2012;125:5944–9. 10.1242/jcs.113100 23038775

[23] Lemaitre B, Hoffmann J. The host defense of Drosophila melanogaster. Annu Rev Immunol. 2007;25:697–743. 17201680 10.1146/annurev.immunol.25.022106.141615

[24] Schneider DS, Ayres JS. Two ways to survive infection: what resistance and tolerance can teach us about treating infectious diseases. Nat Rev Immunol. 2008;8:889–95. 10.1038/nri2432 18927577 PMC4368196

[25] Jiang H, Patel PH, Kohlmaier A, et al Cytokine/Jak/Stat signaling mediates regeneration and homeostasis in the Drosophila midgut. Cell. 2009;137:1343–55. 10.1016/j.cell.2009.05.014 19563763 PMC2753793

[26] Sinenko SA, Mathey-Prevot B. Increased expression of Drosophila tetraspanin, Tsp68C, suppresses the abnormal proliferation of ytr-deficient and Ras/Raf-activated hemocytes. Oncogene. 2004;23:9120–8. 15480416 10.1038/sj.onc.1208156

[27] Ghosh S, Singh A, Mandal S, et al Active hematopoietic hubs in Drosophila adults generate hemocytes and contribute to immune response. Dev Cell. 2015;33:478–88. 10.1016/j.devcel.2015.03.014 25959225 PMC4448147

[28] Babcock DT, Brock AR, Fish GS, et al Circulating blood cells function as a surveillance system for damaged tissue in Drosophila larvae. Proc Natl Acad Sci U S A. 2008;105:10017–22. 10.1073/pnas.0709951105 18632567 PMC2474562

[29] Bach EA, Ekas LA, Ayala-Camargo A, et al GFP reporters detect the activation of the Drosophila JAK/STAT pathway in vivo. Gene Expr Patterns. 2007;7:323–31. 17008134 10.1016/j.modgep.2006.08.003

[30] Buchon N, Broderick NA, Kuraishi T, et al Drosophila EGFR pathway coordinates stem cell proliferation and gut remodeling following infection. BMC Biol. 2010;8:152 10.1186/1741-7007-8-152 21176204 PMC3022776

[31] Buchon N, Broderick NA, Chakrabarti S, et al Invasive and indigenous microbiota impact intestinal stem cell activity through multiple pathways in Drosophila. Genes Dev. 2009;23:2333–44. 10.1101/gad.1827009 19797770 PMC2758745

[32] Jiang H, Edgar BA. EGFR signaling regulates the proliferation of Drosophila adult midgut progenitors. Development. 2009;136:483–93. 10.1242/dev.026955 19141677 PMC2687592

[33] Buchon N, Broderick NA, Poidevin M, et al Drosophila intestinal response to bacterial infection: activation of host defense and stem cell proliferation. Cell Host Microbe. 2009;5:200–11. 10.1016/j.chom.2009.01.003 19218090

[34] Amcheslavsky A, Jiang J, Ip YT. Tissue damage-induced intestinal stem cell division in Drosophila. Cell Stem Cell. 2009;4:49–61. 10.1016/j.stem.2008.10.016 19128792 PMC2659574

[35] Wright VM, Vogt KL, Smythe E, et al Differential activities of the Drosophila JAK/STAT pathway ligands Upd, Upd2 and Upd3. Cell Signal. 2011;23:920–7. 10.1016/j.cellsig.2011.01.020 21262354

[36] Hombria JC, Brown S, Hader S, et al Characterisation of Upd2, a Drosophila JAK/STAT pathway ligand. Dev Biol. 2005;288:420–33. 16277982 10.1016/j.ydbio.2005.09.040

[37] Bunker BD, Nellimoottil TT, Boileau RM, et al The transcriptional response to tumorigenic polarity loss in Drosophila. Elife. 2015;4. 10.7554/eLife.03189 PMC4369581 25719210

[38] Martin-Blanco E, Gampel A, Ring J, et al puckered encodes a phosphatase that mediates a feedback loop regulating JNK activity during dorsal closure in Drosophila. Genes Dev. 1998;12:557–70. 9472024 10.1101/gad.12.4.557 PMC316530

[39] Weber U, Paricio N, Mlodzik M. Jun mediates Frizzled-induced R3/R4 cell fate distinction and planar polarity determination in the Drosophila eye. Development. 2000;127:3619–29. 10903185 10.1242/dev.127.16.3619

[40] Biteau B, Karpac J, Supoyo S, et al Lifespan extension by preserving proliferative homeostasis in Drosophila. PLoS Genet. 2010;6:e1001159 10.1371/journal.pgen.1001159 20976250 PMC2954830

[41] Rera M, Clark RI, Walker DW. Intestinal barrier dysfunction links metabolic and inflammatory markers of aging to death in Drosophila. Proc Natl Acad Sci U S A. 2012;109:21528–33. 10.1073/pnas.1215849110 23236133 PMC3535647

[42] Rera M, Azizi MJ, Walker DW. Organ-specific mediation of lifespan extension: more than a gut feeling? Ageing Res Rev. 2013;12:436–44. 10.1016/j.arr.2012.05.003 22706186 PMC3498542

[43] Ayyaz A, Li H, Jasper H. Haemocytes control stem cell activity in the Drosophila intestine. Nat Cell Biol. 2015;17:736–48. 10.1038/ncb3174 26005834 PMC4449816

[44] Souba WW, Klimberg VS, Plumley DA, et al The role of glutamine in maintaining a healthy gut and supporting the metabolic response to injury and infection. J Surg Res. 1990;48:383–91. 2187115 10.1016/0022-4804(90)90080-l

[45] Yang H, Kronhamn J, Ekstrom JO, et al JAK/STAT signaling in Drosophila muscles controls the cellular immune response against parasitoid infection. EMBO Rep. 2015;16:1664–72. 10.15252/embr.201540277 26412855 PMC4687419

[46] Dostert C, Jouanguy E, Irving P, et al The Jak-STAT signaling pathway is required but not sufficient for the antiviral response of drosophila. Nat Immunol. 2005;6:946–53. 16086017 10.1038/ni1237

[47] Sorrentino RP, Melk JP, Govind S. Genetic analysis of contributions of dorsal group and JAK-Stat92E pathway genes to larval hemocyte concentration and the egg encapsulation response in Drosophila. Genetics. 2004;166:1343–56. 15082553 10.1534/genetics.166.3.1343 PMC1470785

[48] Bahia AC, Kubota MS, Tempone AJ, et al The JAK-STAT pathway controls Plasmodium vivax load in early stages of Anopheles aquasalis infection. PLoS Negl Trop Dis. 2011;5:e1317 10.1371/journal.pntd.0001317 22069502 PMC3206008

[49] Kemp C, Mueller S, Goto A, et al Broad RNA interference-mediated antiviral immunity and virus-specific inducible responses in Drosophila. J Immunol. 2013;190:650–8. 10.4049/jimmunol.1102486 23255357 PMC3538939

[50] Van De Bor V, Zimniak G, Papone L, et al Companion Blood Cells Control Ovarian Stem Cell Niche Microenvironment and Homeostasis. Cell Rep. 2015;13:546–60. 10.1016/j.celrep.2015.09.008 26456819

[51] Woodcock KJ, Kierdorf K, Pouchelon CA, et al. Macrophage-derived upd3 cytokine causes impaired glucose homeostasis and reduced lifespan in Drosophila fed a lipid-rich diet. Immunity. 2015;42:133–44. 25601202 10.1016/j.immuni.2014.12.023 PMC4304720

[52] Nappi A, Poirie M, Carton Y. The role of melanization and cytotoxic by-products in the cellular immune responses of Drosophila against parasitic wasps. Adv Parasitol. 2009;70:99–121. 10.1016/S0065-308X(09)70004-1 19773068

[53] Krautz R, Arefin B, Theopold U. Damage signals in the insect immune response. Front Plant Sci. 2014;5:342 10.3389/fpls.2014.00342 25071815 PMC4093659

[54] Ahrens S, Zelenay S, Sancho D, et al F-actin is an evolutionarily conserved damage-associated molecular pattern recognized by DNGR-1, a receptor for dead cells. Immunity. 2012;36:635–45. 10.1016/j.immuni.2012.03.008 22483800

[55] Razzell W, Evans IR, Martin P, et al Calcium flashes orchestrate the wound inflammatory response through DUOX activation and hydrogen peroxide release. Curr Biol. 2013;23:424–9. 10.1016/j.cub.2013.01.058 23394834 PMC3629559

[56] Brown S, Hu N, Hombria JC. Identification of the first invertebrate interleukin JAK/STAT receptor, the Drosophila gene domeless. Curr Biol. 2001;11:1700–5. 11696329 10.1016/s0960-9822(01)00524-3

[57] Charroux B, Royet J. Elimination of plasmatocytes by targeted apoptosis reveals their role in multiple aspects of the Drosophila immune response Proc Natl Acad Sci USA. 2009;106:9797–802. 10.1073/pnas.0903971106 19482944 PMC2700997

[58] Defaye A, Evans I, Crozatier M, et al Genetic ablation of Drosophila phagocytes reveals their contribution to both development and resistance to bacterial infection. *J* Innate Immun. 2009;1:322–34. 10.1159/000210264 20375589

[59] Basset A, Khush RS, Braun A, et al The phytopathogenic bacteria *Erwinia carotovora* infects *Drosophila* and activates an immune response. Proc Natl Acad Sci U S A. 2000;97:3376–81. 10725405 10.1073/pnas.070357597 PMC16247

[60] Neyen C, Bretscher AJ, Binggeli O, et al Methods to study Drosophila immunity. Methods. 2014;68:116–28. 10.1016/j.ymeth.2014.02.023 24631888

[61] Hudry B, Khadayate S, Miguel-Aliaga I. The sexual identity of adult intestinal stem cells controls organ size and plasticity. Nature. 2016;530:344–8. 10.1038/nature16953 26887495 PMC4800002

