# Supplementary figures and images for: Remote Control of Intestinal Stem Cell Activity by Haemocytes in Drosophila
Source: PLoS Genet. 2016 May 27;12(5):e1006089. doi: 10.1371/journal.pgen.1006089 (PMC4883764; doi:10.1371/journal.pgen.1006089)

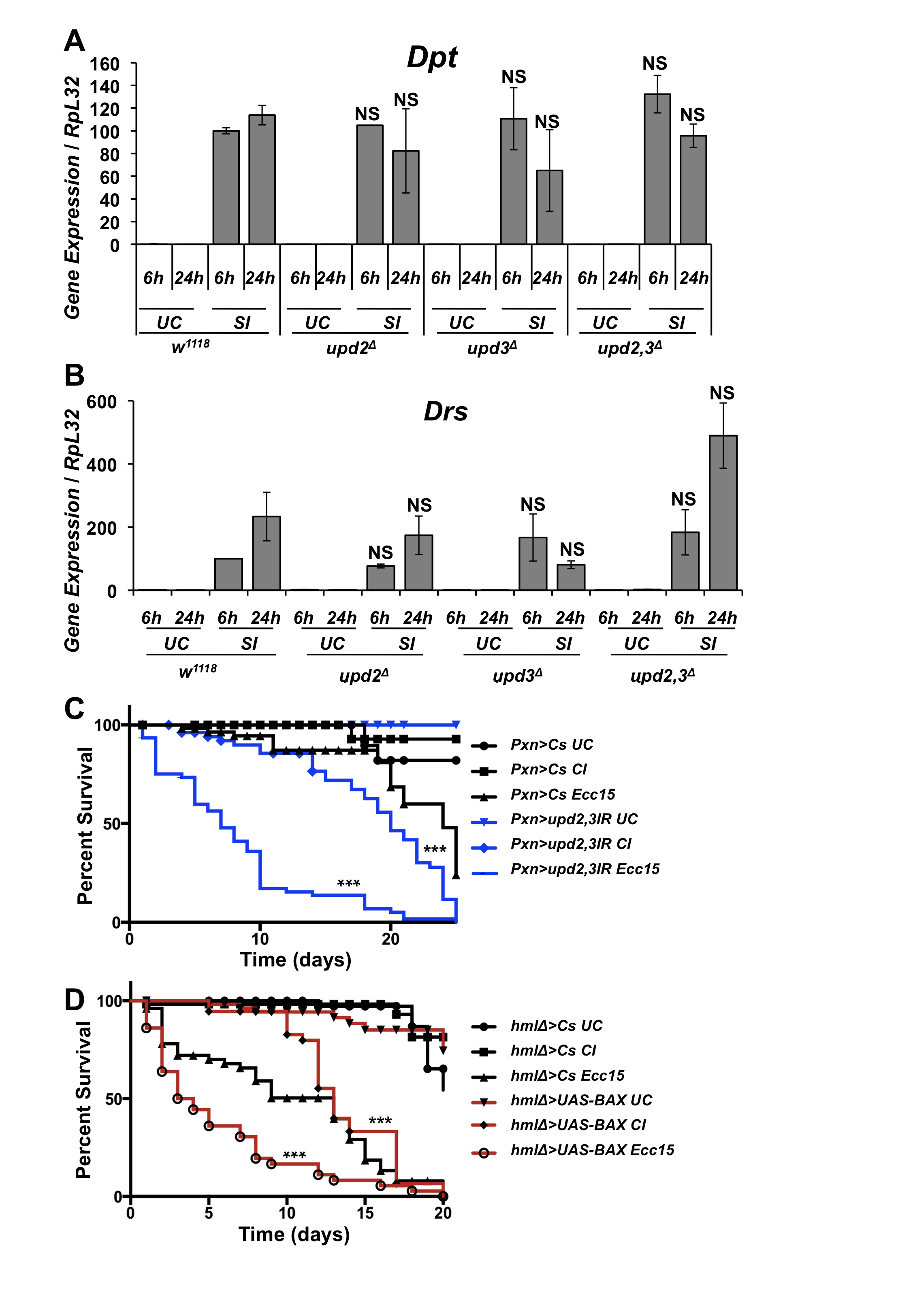

Supplement: S1 Fig — (A and B) RT-qPCR analysis of Diptericin (Dpt) and Drosomycin (Drs) expression in adults either unchallenged (UC) or collected at 6 h and 24 h after septic injury with Ecc15 (A) or M. luteus (B). Data are the mean of three repeats ± SE. The P value was determined NS (non-significant) using pair-wise comparison between each time-point of upd2Δ, upd3Δ and upd2,3Δ infected to w1118 infected using Student’s t-test. (C) Knockdown of both upd2 and upd3 in haemocytes using the PxnGAL4 driver leads to a higher mortality rate in flies subjected to either clean injury or septic injury with Ecc15. Data is pooled from three independent experiments, n = 60. (D) ‘Hemoless’ flies (hmlΔGAL4 > UAS-BAX) display an increased susceptibility to wounding as well as septic injury with Ecc15. Data is pooled from three independent experiments, n = 60. The log-rank test was used to determine statistical significance. P value < 0.001 = *** as determined by log–rank test for hmlΔGAL4 > UAS-BAX CI and Ecc15 SI as compared to hmlΔGAL4 > Cs CI and Ecc15 SI; P value < 0.001 = *** for pxnGAL4 > UAS-upd2,3IR CI and Ecc15 SI compared to pxnGAL4> Cs CI and Ecc15 SI. Male flies were used for experiments done in panels A-D. (TIF) [file pgen.1006089.s001.tif]

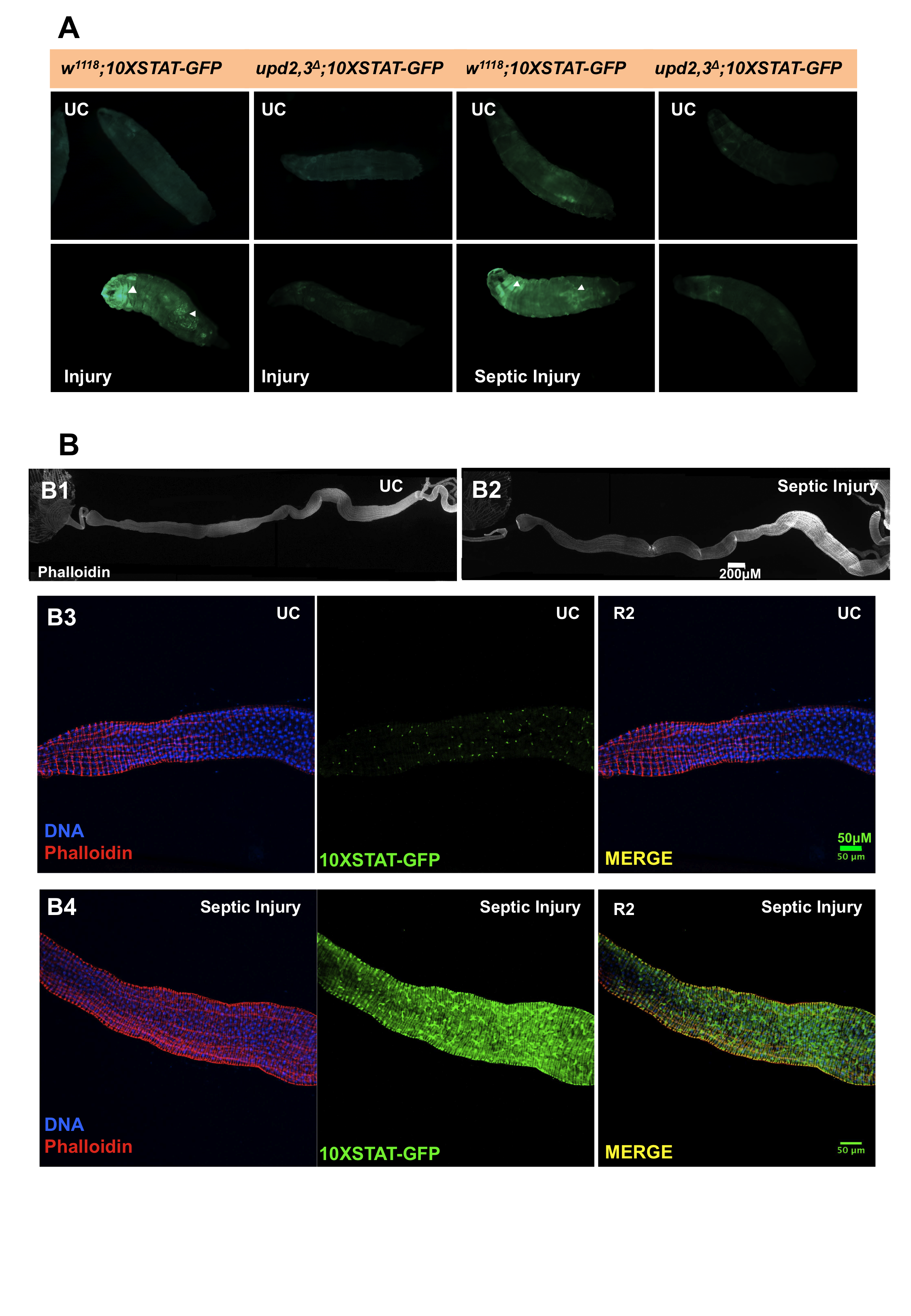

Supplement: S2 Fig — (A) A 10XSTAT-GFP (green) reporter allele was used to monitor JAK/STAT activity in a living third-instar larva. Live-imaging of larvae at 6 h post-injury revealed an increase of GFP signal in the fat body and the gut. No increase in GFP signal was observed in the upd2,3Δ larvae upon injury. Representative images from unchallenged (UC) flies or flies collected 6 h after clean injury or septic injury. The white arrowheads in wild-type clean injury and septic injury indicate GFP signal in the gut of larvae while white arrows indicate GFP in the fat body of the head. (B1-B4) Immunostaining on 10XSTAT-GFP (green) reporter gene and visceral muscle using Rhodamine-phalloidin (red) in the intestine region R2 of adult female flies either unchallenged (UC) or collected 6 h after septic injury with Ecc15. Nuclei are stained using DAPI (blue) and co-staining of 10XSTAT-GFP expression with the visceral muscle is seen in yellow. (TIF) [file pgen.1006089.s002.tif]

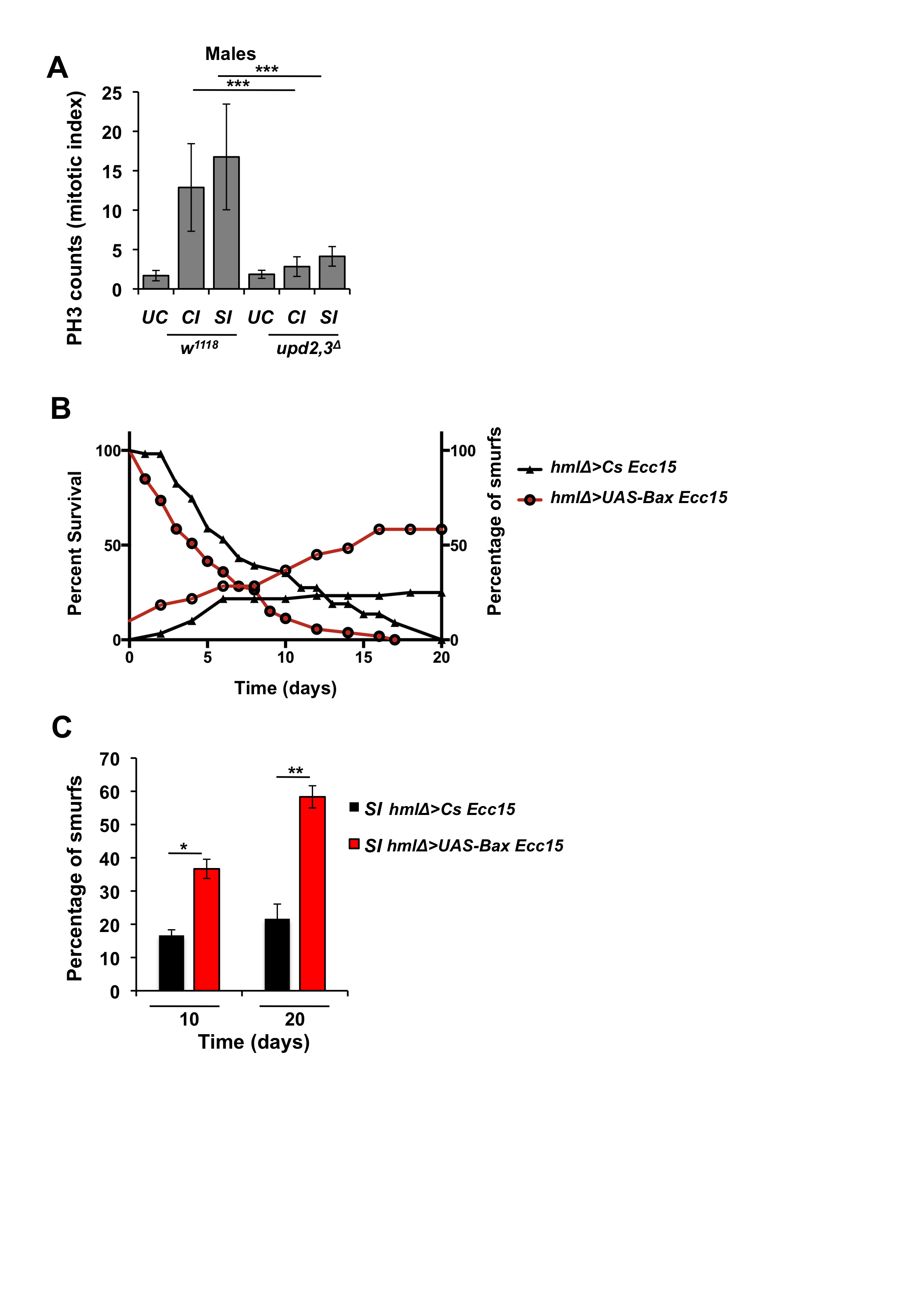

Supplement: S3 Fig — (A) The mitotic index of upd2,3Δ male flies as compared to the w1118 in the midgut of unchallenged flies (UC) and in flies subjected to clean injury (CI) or septic injury (SI). Males show a reduced intestinal stem-cell proliferation as compared to females after injury and septic infection (Compare Figs 5A and S3A, [61]). (B) Smurf assay and survival curves for hmlΔGAL4 > UAS-Bax Ecc15 SI. The right Y-axis curve is the cumulative proportion of Smurfs in the population. hmlΔGAL4 > UAS-Bax flies display higher proportion of Smurf phenotype after septic injury (SI) as compared to their wild-type counterparts. (C) Proportions Smurfs in hmlΔGAL4 > Cs SI male flies and hmlΔGAL4 > UAS-Bax Ecc15 SI male flies at day 10 and day 20 after infection. 60 flies/condition were used, and **: p< 0.01, *: p< 0.05 as determined by Student’s t-test. (TIF) [file pgen.1006089.s003.tif]

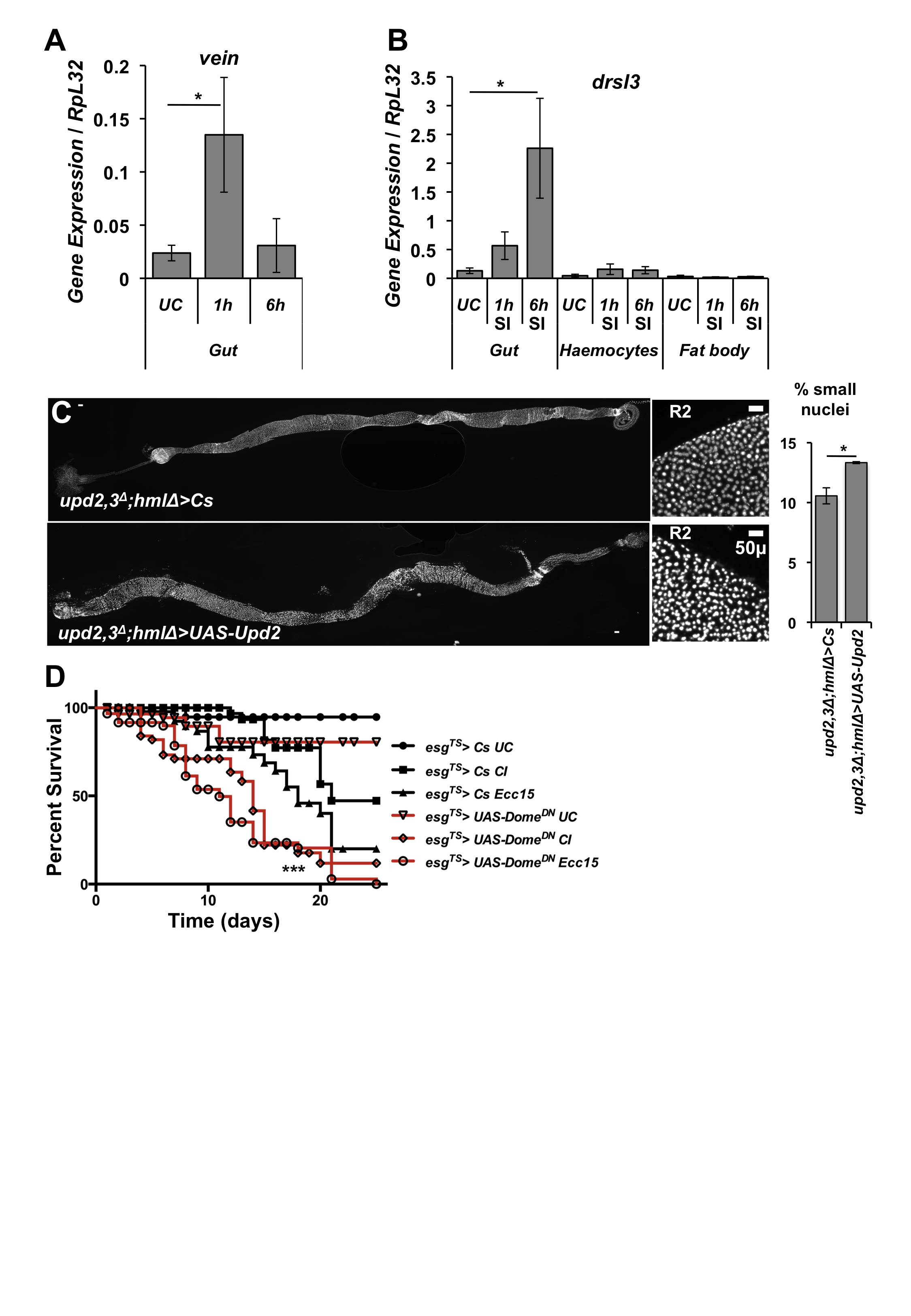

Supplement: S4 Fig — (A) The expression of vein was measured 1 h and 6 h after septic injury. (B) RT-qPCR experiments show that the putative antimicrobial peptide gene Drsl3 is induced in the intestine upon septic injury. (C) Over-expression of Upd2 in haemocytes in upd2,3Δ flies(genotype: upd2,3Δ, UAS-upd2/+; hmlΔGAL4/+) leads to the accumulation of small nuclei cells in the adult intestine as revealed by DAPI staining, pointing to a defect in differentiation. (D) Reducing JAK/STAT signaling by over-expression of a dominant-negative form of the receptor Domeless (UAS-DomeDN) using the intestinal progenitor specific driver escargotTS, leads to an increased mortality in male flies subjected to either a clean injury or a septic injury. Data is pooled from four independent experiments. The log-rank test was used to determine statistical significance. P value < 0.001 = *** as determined by log–rank test for esgTS-GAL4; UAS-DomeDN CI and Ecc15 SI as compared to esgTS-GAL4; Cs CI and Ecc15 SI. (TIF) [file pgen.1006089.s004.tif]

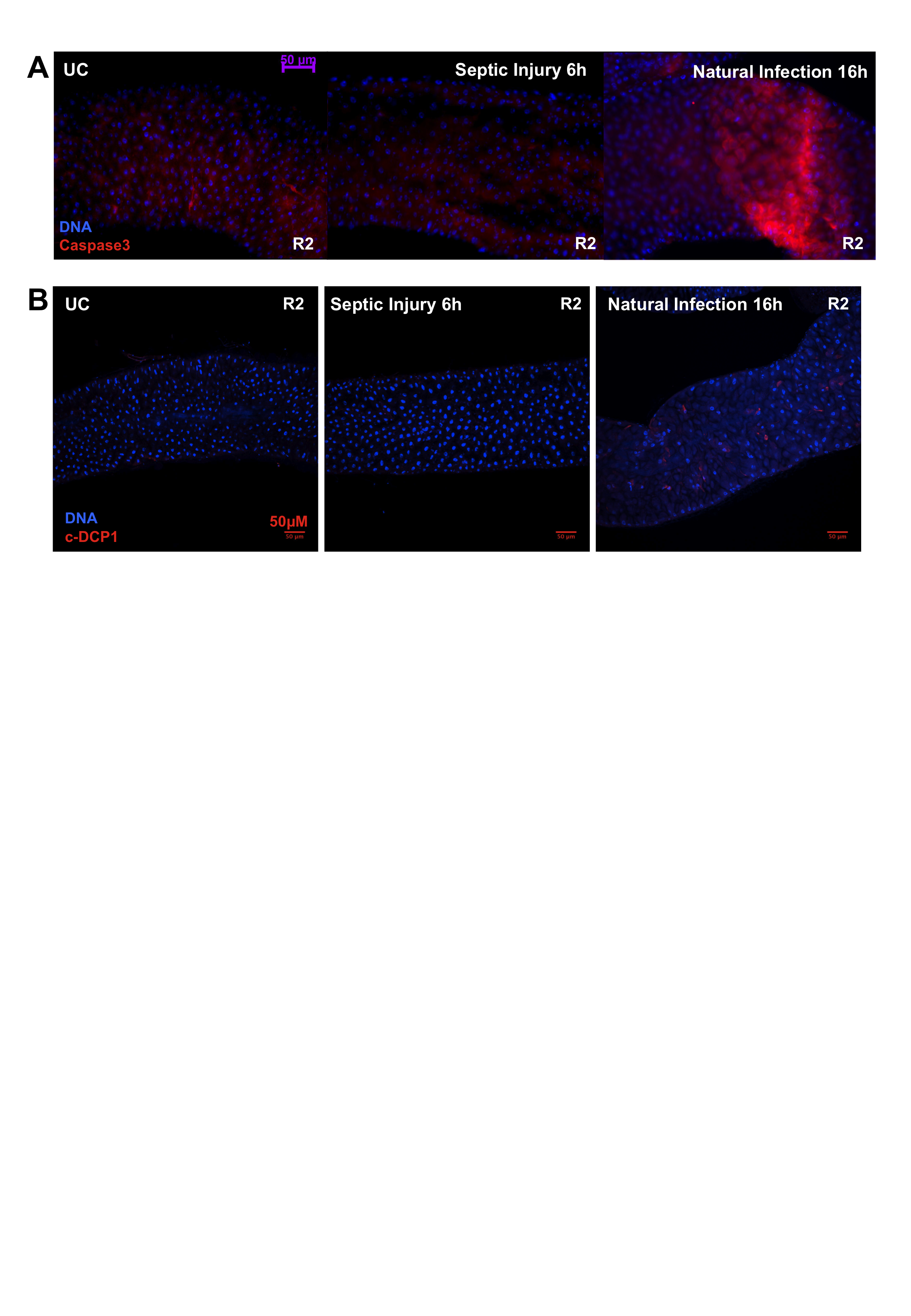

Supplement: S5 Fig — (A-B) Immunostaining using antibodies directed against activated Caspase 3 (A) and cleaved DCP-1 (B) shows that oral infection with Ecc15 but not septic injury induces an increase the level of apoptosis. Shown is the R2 region of female midgut from flies either unchallenged (UC) or collected 6 h after septic injury and 16 h after natural infection with Ecc15. Nuclei are stained using DAPI (blue) and co-staining of Caspase 3 in red. (TIF) [file pgen.1006089.s005.tif]

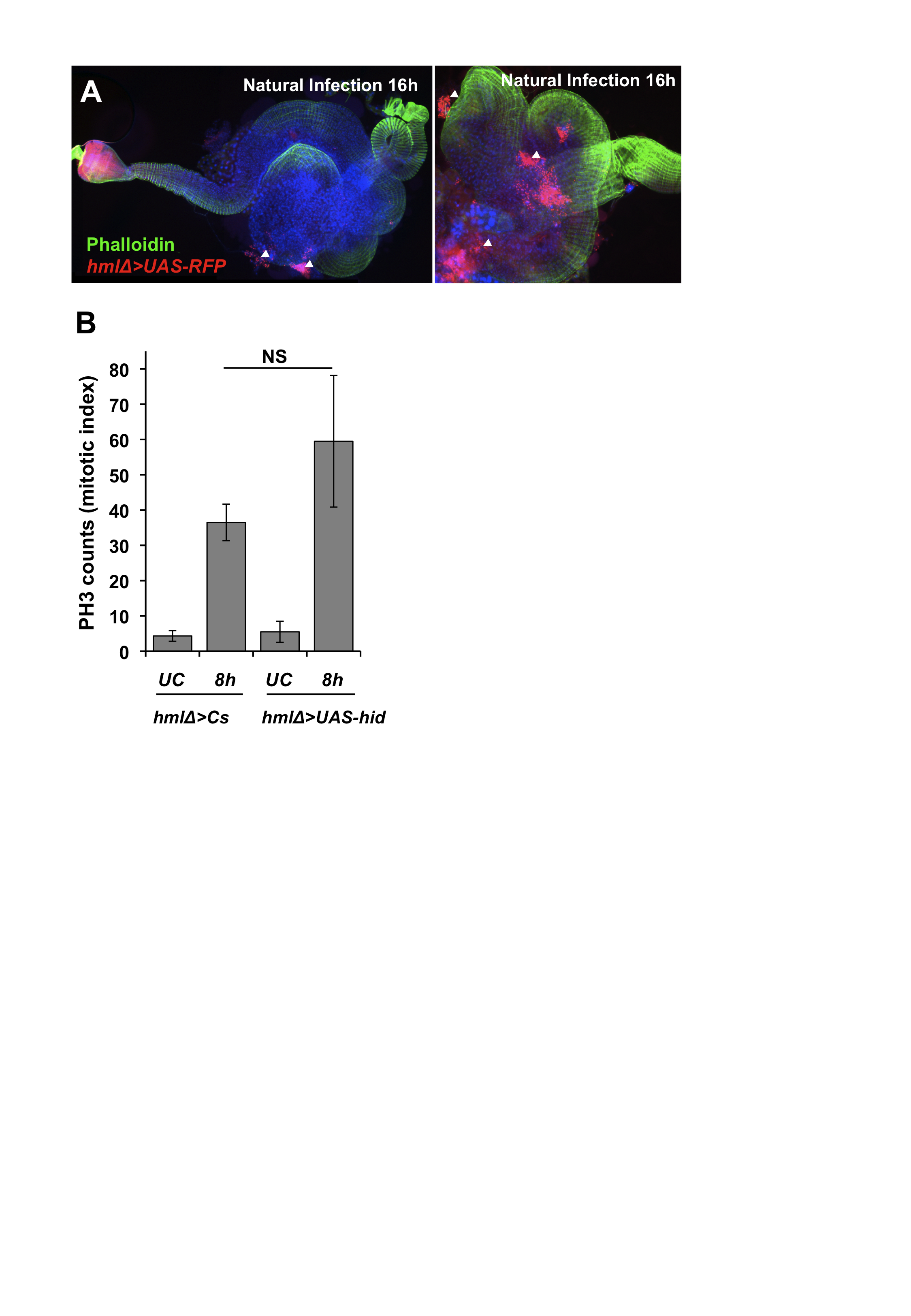

Supplement: S6 Fig — (A) Visualization of gut-associated haemocytes using hmlΔGAL4>UAS-RFP flies. Intestines were fixed with 4% paraformaldehyde and stained with FITC-phalloidin to label visceral muscle and DAPI to label nuclei. While the number of haemocytes attached to the loop of the midgut was variable, we did not observe any marked variation in haemocytes number upon septic injury. (B) ‘Hemoless’ female flies due to the overexpression of the pro-aptototic gene hid in hemocytes have wild-type level of intestinal stem cell proliferation 8 h after oral infection with Ecc15 as determined by counting PH3+ cells. Genotype: hmlΔGAL4 > UAS-hid. (TIF) [file pgen.1006089.s006.tif]
