# Supplementary material for: Remote Control of Intestinal Stem Cell Activity by Haemocytes in Drosophila
Source: PLoS Genet. 2016 May 27;12(5):e1006089. doi: 10.1371/journal.pgen.1006089 (PMC4883764; doi:10.1371/journal.pgen.1006089)
Supplement: S1 Table — (DOCX) [file pgen.1006089.s007.docx]

**Table 1: List of qPCR primer sequences used in this study**

| **Gene** | **Left Primer 5’ to 3’** | **Right Primer 5’ to 3’** |
| --- | --- | --- |
| *RpL32* | GACGCTTCAAGGGACAGTATCTG | AAACGCGGTTCTGCATGAG |
| *upd* | AATCAGCTGAAGCGCCACG | GGAATTGGGCTTGAGCTTGG |
| *upd2* | GGCTCTTCTGCTGATCCTTG | AAGACTTGGTACCGCCACAT |
| *upd3* | GCGGGGAGGATGTACC | GTCTTCATGGAATGAGCC |
| *Drs* | CGTGAGAACCTTTTCCAATATGAT | TCCCAGGACCACCAGCAT |
| *drsl3* | CAGTTGTCATGGTGCAGATGATATTCC | GGCACGTAAGCATTGCCTTTCAGG |
| *Socs36e* | GCACAGAAGGCAGACC | ACGTAGGAGACCCGTAT |
| *Dpt* | GCTGCGCAATCGCTTCTACT | TGGTGGAGTGGGCTTCATG |
| *TotA* | CTGCTCTTATGTAAGTAGTATCGAAT | CAACGATCCTCGCCTTTCGACC |
| *TotM* | TCGACAGCCTGGTCACTTTC | ACCAAGACCACACGAGCATT |
| *vein* | AGCCCATGAGGGTCAC | AGCTCGGAACGTCTTT |
